# Elongation, proliferation & migration differentiate endothelial cell phenotypes and determine capillary sprouting

**DOI:** 10.1186/1752-0509-3-13

**Published:** 2009-01-26

**Authors:** Amina A Qutub, Aleksander S Popel

**Affiliations:** 1 Department of Biomedical Engineering, School of Medicine, Johns Hopkins University, 720 Rutland Avenue, Baltimore, MD 21205, USA

## Abstract

**Background:**

Angiogenesis, the growth of capillaries from preexisting blood vessels, has been extensively studied experimentally over the past thirty years. Molecular insights from these studies have lead to therapies for cancer, macular degeneration and ischemia. In parallel, mathematical models of angiogenesis have helped characterize a broader view of capillary network formation and have suggested new directions for experimental pursuit. We developed a computational model that bridges the gap between these two perspectives, and addresses a remaining question in angiogenic sprouting: how do the processes of endothelial cell elongation, migration and proliferation contribute to vessel formation?

**Results:**

We present a multiscale systems model that closely simulates the mechanisms underlying sprouting at the onset of angiogenesis. Designed by agent-based programming, the model uses logical rules to guide the behavior of individual endothelial cells and segments of cells. The activation, proliferation, and movement of these cells lead to capillary growth in three dimensions. By this means, a novel capillary network emerges out of combinatorially complex interactions of single cells. Rules and parameter ranges are based on literature data on endothelial cell behavior in vitro. The model is designed generally, and will subsequently be applied to represent species-specific, tissue-specific in vitro and in vivo conditions.

Initial results predict tip cell activation, stalk cell development and sprout formation as a function of local vascular endothelial growth factor concentrations and the Delta-like 4 Notch ligand, as it might occur in a three-dimensional in vitro setting. Results demonstrate the differential effects of ligand concentrations, cell movement and proliferation on sprouting and directional persistence.

**Conclusion:**

This systems biology model offers a paradigm closely related to biological phenomena and highlights previously unexplored interactions of cell elongation, migration and proliferation as a function of ligand concentration, giving insight into key cellular mechanisms driving angiogenesis.

## Background

As a new capillary grows from a blood vessel, a series of cellular processes occur. These vascularization events have been extensively studied experimentally, however the whole angiogenic sequence has yet to be characterized by any experiment or model, and numerous unknowns remain. What is known is that an endothelial cell from an existing vessel becomes activated. The activated cell starts to migrate into the extracellular matrix by degrading it; this unique, spindle-shaped cell is called the tip cell. Cells adjacent to the tip cell begin to proliferate, and follow the tip cell; they are referred to as stalk cells. These processes result in formation of a sprout [1]. This capillary sprout moves towards a stimulus, in response to chemical cues, mechanical factors, and a degree of random motility. Finally, the sprout joins an adjacent capillary. Together these events define the process of sprouting angiogenesis.

Hypoxia is a main stimulus for angiogenesis during ischemia, exercise, inflammation, and cancer. In response to hypoxia, a transcription factor hypoxia-inducible factor 1 (HIF1) activates hundreds of genes in cells exposed to low oxygen [2,3]. These genes include vascular endothelial growth factor, VEGF. Overall, VEGF protein stimulates chemotaxis and proliferation of endothelial cells. There are seven known isoforms of VEGF, each with a different effect on cell behavior, and ultimately, on vascular pattern formation [4,5]; additionally, there are splice variants of the VEGF isoforms, VEGF_xxx_b [6]. Here, we first refer to HIF1-dependent expression of VEGF and represent the effect of the VEGF-A (VEGF_165_) isoform on cells, unless otherwise specified.

Along with VEGF, another ligand, Delta, and its receptor play a key role in angiogenic tip cell formation and proliferation, and the integrity of a microvascular network. Recent studies have focused on the multiple effects of Notch-Delta signaling on vascular sprout formation. Delta-like ligand 4 (Dll4) is a transmembrane ligand for Notch receptors, and it is critical to vascular development. So important is Dll4, that like VEGF, haploinsufficiency of the Dll4 gene is embryonically lethal in many mouse strains, as a result of extensive vascular defects [7-9]. Dll4 is primarily expressed in endothelial cells, and correlated to the local concentration of VEGF [10], as well as VEGF receptor concentrations. A blockade of VEGF leads to a decrease of Dll4 [11], while Notch-Delta signaling downregulates VEGFR2 [12]. One study showed the presence of Dll4 reduced tip cell formation as a function of VEGF [13], and another demonstrated Notch suppressed branching and proliferation at the sprout tip [7]. A Dll4 deficiency causes an increase in sprout formation but vessels appear nonproductive, with less capability of carrying blood or reducing hypoxia in surrounding tissue [11]. Overexpression of Dll4 diminishes the growth of new sprout tips. In the computational research presented here, we focus on the effects of VEGF protein concentrations and Dll4 haploinsufficiency on endothelial cells and how this cell level behavior contributes to differences in capillary network formation.

Mathematical representations of angiogenesis date to the 1970's, and their numbers continue to expand rapidly. Some of the first models were differential equations representing a generic growth factor as a chemotactic stimulus, produced and released by a tumor mass, and inducing growth of vessels into the tumor [14-16]. Models have since included detailed equation-based network models of tumor-induced angiogenesis [17], a model of capillary growth through a corneal pocket assay [18], molecular level interactions of VEGF complexes coupled to vessel oxygenation [19], a cell-level rule-based model of network growth in mesenteric tissue [20], Potts models of angiogenic growth [21,22], a model of tip cell selection as a function of notch-signaling [23], network formation stemming from capillary movement through a matrix composed of aligned collagen fibers [24], and VEGF-driven angiogenic growth applied to a vascular engineering construct environment [25], among many others [26-30]. Each model brings a new perspective on the biological phenomenon behind neovascularization, and together they give insight on multiple conditions affecting angiogenesis, and multiple conditions affected by angiogenesis. The goal of the current modeling effort is to provide a framework where many of these models could be employed or integrated, or at the very least their hypotheses tested in a new context, specifically related to biological observations and experimental data.

For this reason, we chose to design the model using the broad framework of three-dimensional agent-based modeling. Agent-based programming has roots in social science, game theory, economics, evolution and public health. More recently it has emerged as a tool useful for a range of biomedical applications, including angiogenesis [20], membrane transport [31,32], inflammatory response [33-35], and tumor growth [36,37].

Agents are objects that can interact with their environment, and modify their surroundings. They are analogous to digital organisms familiar to evolutionary biology in that they carry a computational genome, or a sequence of instructions (henceforth called rules). These rules determine the agents' response to logical functions. The rules of agents require listing the factors that influence cell behavior as events, with direct counterparts in biology. Unlike digital organisms, agents used in the model are not inherently self-replicating. Agents' rules may evolve, and they may copy their instructions when modified to represent growth.

In agent-based modeling, global functions (e.g., global ischemia) and sophisticated rules can govern agent behavior. Agent interactions with one another and their environment can also be asynchronous. The rule-based modeling we describe is a continual, iterative process, much like perfecting in vitro or in vivo experiments. As more knowledge is gained, the current assumptions may change, and a cycle of improvements is needed to keep pace with current biological information. Furthermore, we start with a very general model – its parameters will be changed to represent specific species, tissues and conditions.

We employed this agent-based approach to develop a three-dimensional, computational model that simulates cellular sprouting at the onset of angiogenesis. We use the model to determine and weigh the critical events in angiogenesis; and differentiate under what microenvironments, which factors dominate and result in a particular vessel and capillary network phenotype.

The model is based on experimental work found from extensive literature research, and methods in the model are closely governed by biological mechanisms. Currently, the model is applied to conditions that might occur in a three-dimensional in vitro setting. We represent physiological changes at the cell level; visually simulate in three dimensions assumptions behind cell activation, migration, elongation, proliferation and branching; and test cell level behavior in response to different stimuli, focusing in the current model on activation by a threshold change in VEGF and changes in ligand presence. A novel capillary network emerges out of this complex interaction of single cells.

Results of the model show the relationship between growth factor gradients, cell sprouting, cell migration and cell proliferation. Results also showed how variations in the mechanisms of cellular persistence alter vessel growth. We predicted the effects of migration separate from proliferation on tip cell and stalk cell movement, and on total vessel growth. Furthermore, the model represents novel findings of how Delta ligand changes influence capillary phenotype. Overall, the model represents a three-dimensional framework upon which to test and develop biologically realistic mechanisms underlying blood vessel growth.

## Methods

### Model Formulation

Model inputs are coordinates of an initial 3D vascular network, local VEGF concentrations, binary values for five parameters (proliferation of tip cells, proliferation of stalk cells, tip migration, elongation, Dll4 presence), and initial values of variables. Output is the resulting change in cell activation, cell position, cell growth and vessel phenotype after the series of biologically-based rules determine cells' response to the local environment. Rules are implemented through logical statements and equations. Rules and parameter ranges are initially based on available literature data on endothelial cell behavior in vitro (Tables 1, 2, 3). While this version focuses solely on endothelial cells, subsequent iterations of the model can include other cell types important to different angiogenic processes, e.g., smooth muscle cells, pericytes, precursor cells and astrocytes.

**Table 1:** Model parameters and their abbreviations.

**Variable**	**Abbreviation**
Concentration of A (ng/ml)	[A]
Hypoxia inducible factor (HIF1α)	Hα
Vascular endothelial growth factor	VEGF
Matrix metalloproteinase	MMP
Notch ligand Delta-like 4	Dll4
Gradient (concentration of species A) (ng/ml/μm)	∇A
Standard deviation	σ
Probability distribution	ϕ
Cell position	X(i,j,k)
Velocity (μm/s)	ν(i,j,k)
Directional vector	**d**
Persistence	p
Degree of randomness	μ
Length (μm)	ℓ
Time (hr)	t
Elongation constant	*ε*
Total tip cell movement	m_total_
Migration of the tip cell (μm)	M_tip_
Elongation of the tip cell (μm)	E_tip_
Elongation of the adjacent stalk cell segment (μm)	E_stalk_
Proliferation of tip cell (% volume increase)	P_tip_
Proliferation of stalk cell (% volume increase)	P_stalk_
Cell volume (μm^3^)	V
Total stalk cell volume in a sprout (μm^3^)	V_sproutStalk_
Radius of cell (μm)	R
Radius after proliferation (μm)	R_P_
Outer radius of existing capillary (μm)	R_cap_
Radius of inner lumen in existing capillary (μm)	r_lumen_
Length of tip cell (μm)	ℓ_tip_
Length of adjacent stalk cell segment (μm)	ℓ_Stalk_
Length due to stalk cell growth (μm)	$\ell_{{\text{P}}_{\text{stalk}}}$
Length due to tip cell growth (μm)	$\ell_{{\text{P}}_{\text{tip}}}$
Length due to stalk cell stretching (μm)	$\ell_{{\text{E}}_{\text{stalk}}}$
Random number generator	rgen
Grid height (μm)	g_H_
Grid width (μm)	g_W_
Grid length (μm)	g_l_

**Table 2:** Parameters for the cell model.

**Parameter**	**Value**	**Reference**
Default [VEGF]_0_	0.20 ng/ml, uniform in grid space unless a VEGF gradient is specified	-
Vessel size	Diameter = 3–14 μm	[84,85]
Initial vessel length	400 μm (13–2000 μm references)	[1,42,86-89]
Initial cell size in vessel	Diameter: 4 μm (3–14 μm)	[90,91]
	Length: 100 μm (20–107 μm)	
Initial tip cell length	5 μm	-
Initial radius of tip cell	1 μm	-
Initial length of stalk cells	0 μm	-
Initial radius of formed stalk cells	2 μm	-
Average distance between initial capillaries	20 μm (20–40 μm)	In skeletal muscle: [92]
		In brain tissue: [93]
Initial ratio of stalk cell radius to stalk cell length	0.05–0.1	-
Number of initial endothelial cells per capillary	4 cells (2–6) cells	[85,86,90]
Number of activated cells adjacent to tip cell	1–2 cells	-
Initial branch length	0–4.2 μm (minimum non-zero branch length of 1.4 μm growth in one time-step of 2 hrs)	[69,85,88,89]
Branch angle	0–120°	[94]
Maximum elongation of stalk cells	ε_max _= 0.5; maximum elongation length is 1.5 ℓ_stalk _(physiologically, different stimuli cause an increase of 0.2–1.8× average length)	Addition of EGF; cyclic mechanical stretch [53,95]
Maximum elongation of tip cells	ε_max _= 0.5; maximum elongation length is 1.5 ℓ_tip_	[53]
Maximum velocity for a cell in three-dimensions	7.5 μm/hr	
Radius of lumen (r_lumen_)	Constant; range 1–4 μm	-
Volume of stalks cells	$\text{V}=\pi \cdot ({\text{R}}^{2})\cdot {\ell_{\text{stalk}}}^{\mu {\text{m}}^{3}}$	-
Volume of tip cells	$\text{V}=\pi \cdot ({\text{R}}^{2})\cdot {\ell_{\text{tip}}}^{\mu {\text{m}}^{3}}$	-
Volume of stalk cells in capillary	$\text{V}=\pi \cdot ({\text{R}}_{\text{cap}}^{\text{2}}-{\text{r}}_{\text{lumen}}^{2})\cdot {\ell_{\text{stalk}}}^{\mu {\text{m}}^{3}}$	-
Cell length change as a function of volume change, where radius to length ratio is held constant	ℓ^3 ^≈ volume	-

Values are experimentally determined or estimated. The default value used in the model is given first; value ranges found in references and used for sensitivity analysis are provided in parentheses.

**Table 3:** Rules and related experimental references for endothelial cell sprouting.

**Rule**	**Logical or Mathematical Statement**	**Reference**
**Tip Cell Activation**	[VEGF] > 0.5 ng/ml, and vacancy in environment surrounding the tip cell	[44]

**VEGF gradient, global ∇VEGF**	Variable.	-
	Default gradient: [VEGF] (ng/ml) in each voxel is uniform, except within a restricted volume. Within this volume, it is randomly generated at the start of each model, and dependent on location.The probability distribution for [VEGF] at location X(i,j,k) is defined by:	
	$\phi ({\left[\text{VEGF}\right]}_{\text{j}})=\{\frac{1}{\sqrt{2\pi \sigma }}\exp\left[-{\left(\frac{{\left[\text{VEGF}\right]}_{\text{j}}-{\left[\text{VEGF}\right]}_{\text{mean},\text{j}}}{\sqrt{2}\sigma }\right)}^{2}\right]$	
	• where g_w_/W2 < i < g_w_/W1 ${\left[\text{VEGF}\right]}_{\text{meanj}}=\left|\text{j}\cdot \frac{\text{C}1}{{\text{g}}_{\text{h}}}-\text{C}2\right|$	
	• where j > g_h_/H1	
	• where g_l_·L2 < k < g_l_·L1	
	• σ = C3·[VEGF]_mean,j_	

**Unrestricted Tip Cell Migration Rate with VEGF, M_tip_**	T1·[VEGF (in ng/ml)] + migNoVEGF μm/hr	[54,56,57,96]

**Cell Migration Rate without VEGF, migNoVEGF**	Default: 6.2 μm/hr	[62,97]
	Physiological Range: 5–11 μm/hr^1^	

**Tip Cell Migration Rate as a Function of Extracellular Matrix Composition and VEGF, M_tip_**	T2·[VEGF (in ng/ml)] + T3·K (fraction collagen content) + migNoVEGFMatrix μm/hr	[46,57,59,62,98]

**Cell Migration Rate without VEGF and minimal/no Matrix, migNoVEGFMatrix**	Default: 1.2 μm/hr	[52]
	Physiological Range: 1.2–30 μm/hr (collagen IV, 2D to glass, 2D)	

**Stalk Cell Proliferation with VEGF, P_stalk_**	% Cell Proliferation vs. Control = P1·[VEGF (in ng/ml)]+ proNoVEGF after 48 to 72 hours (approximate average = 60 hrs)	[10,44,57-59,99,100]

**Initial Tip Cell Growth**	If tip cell is < tipMin in length and no stalk cells are present, tip cell grows to tipMin in current timestep. Thereafter it follows default rules for migration, elongation and proliferation.	-

**Tip Cell Proliferation and Dll4^+/- ^Effect on Tip Proliferation, P_tip_**	For Dll4 +/+, tip cell proliferates at a rate of P_stalk _with 3% probability	[10,13,69,88]
	For Dll4 +/-, tip cell proliferates at a rate of P_stalk _with 8% probability	

**Tip Cell Division**	If tip cell is > tipMax in length, tip cell divides into two cells. The leading cell remains a tip cell, while the cell adjacent to the stalk cells takes on the stalk cell phenotype and rules.	-

**Dll4^+/- ^Effect on Tip Cell Formation**	For Dll4^+/-^, maximum number of tip cells formed per existing capillary of 400 μm length is 2.	[13]
	For Dll4^+/+^, maximum number of tip cells formed per existing capillary of 400 μm length is 1.	

**Dll4^+/- ^Effect on Branching**	For Dll4^+/-^, branchCells = 0.4 and branchTipCells = 0.4. [VEGF] threshold for new tip cell does not need to be crossed. VEGF_branch = 0 ng/ml.	[13]
	For Dll4^+/+^, branchCells = 0.2 and branchTipCells = 0. VEGF_branch = 0.5 ng/ml.	

**Persistence as a Function of [VEGF]**	Weight for a cell's local search is biased in the direction of the global [VEGF] gradient.	[96] The effects of EGF (epithelial growth factor) on persistence was studied in this reference.
	• When local [VEGF] gradients are equal in all directions, the weighing range explored: dirBias/denomBias·[VEGF], where dirBias = 0 to 10 in one direction, where there are eight restricted directions. See Figure 6.	

Below we introduce the model, describe individual rules for cell activity, and explain how the rules work in a discrete grid. A list of model abbreviations and parameters can be found in Tables 1 and 2, respectively, while initial values for variables are shown in Table S1 (see Additional file 1) and rules are listed in Table 3.

### Geometry

At the beginning of each run of the model, the simulation environment is populated by an initial preexisting capillary network. In this rendition, the dimensions of the capillary network are similar to those simulated from rat extensor digitorum longus (rat skeletal muscle), as in reference [38]. For the purpose of showing the initial steps in sprouting, several capillaries (two to four capillaries) were selected randomly from this network. These capillaries are represented in the model as connected endothelial cells. The location and movement of cells are defined in a Cartesian grid, however the methods are portable to other geometries. There are no inherent size restrictions on the space modeled. In this model version, the specific grid dimensions for the program showing two initial capillaries is 20 μm by 20 μm by 400 μm (160,000 cube-shaped voxels of 1 μm^3^), and the grid size shown for three capillaries expands to 100 μm by 100 μm by 400 μm. The k-axis is scaled down by 1/10^th^, as a visual aid. Voxels in the model are occupied by part of the vasculature, or by the matrix and interstitial fluid surrounding the vasculature. Each voxel is associated with a computational datastructure that is capable of storing and passing information in vectors. In the current model, the voxel-associated datastructure contains information on local concentrations of growth factors.

### Representation of Cells

**Figure 1:**
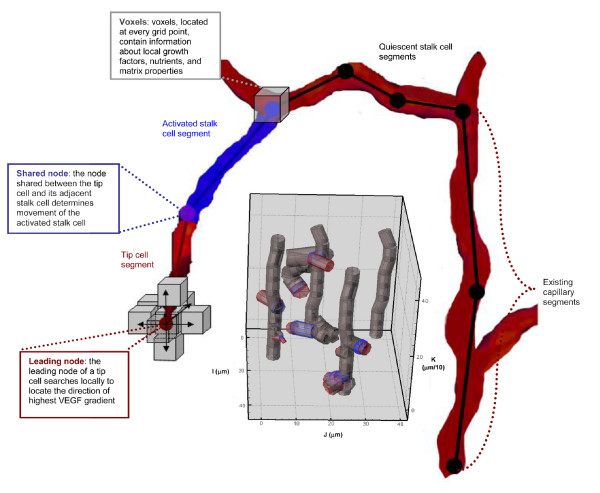
**Schematic of the three-dimensional model**. Capillaries are represented by endothelial cells. An example of a growing network with four capillaries is shown in the gray inset. Cells are divided into segments. Each segment is represented by two nodes. Currently, cell segments are modeled as cylinders specified by a length and radius (gray inset); an activated segment's length and radius can change during a model run. The local environment surrounding a cell is defined in each voxel of the grid. In the present model, voxels contain values for the local VEGF concentration. All cell segments have the capability of sensing what is located in the 26 voxels surrounding each of its nodes. For every timestep of the current model, this sensing is restricted to the leading node of the tip cell (red) and the adjacent node (purple), shared by the tip and activated stalk segment. The local search for the highest growth factor gradient surrounding the leading node of a tip cell determines the direction the sprout tip moves.

Each preexisting capillary vessel is composed of four endothelial cells. Endothelial cells are represented as a series of segments that occupy a cylindrical volume specified by a radius and length (Figure 1, gray inset). Segments are defined by two connected nodes (Figure 1). Each node is associated with a voxel, and serves as a position used to calculate the local environment of a cell segment. An activated tip cell is defined throughout the simulation by one segment (two nodes) that can vary in length, as the cell changes position, grows or elongates. Stalk cells are represented by one activated segment adjacent to the tip cell and by any number of nonadjacent, quiescent segments. As the adjacent stalk cells change position and shape, their segments can change in number and radius, and the activated segment closest to the tip cell can change in length and radius. Endothelial cells on the preexisting capillaries have a static length and radius, throughout a model run.

### Local VEGF Levels

VEGF gradient, ∇[VEGF], and local VEGF concentrations, [VEGF], are inputs into the current model and remain constant for every run of the simulation. This condition can be relaxed by coupling the cell model to previously developed VEGF models [39,40]. For graphs and model runs presented here, there is either no gradient (a uniform [VEGF] throughout the gridspace), or the ∇[VEGF] is defined as in Appendix 1, where noted.

### Rules

Behavioral rules based on biophysical properties and experimental observations govern the activation and movement of endothelial cells in the model. Table 3 lists the main rules governing endothelial cell migration and proliferation, and the related experimental references. To represent biological mechanisms in the model and perform these rules, the computer code implements over 80 logical statements at each timestep, for each active cell segment.

At the beginning of each run of the simulation, Boolean rules are defined. These rules determine proliferation, migration and elongation; see Table 4 (ProliferationTipOn, ProliferationStalkOn, MigrationTipOn, ElongationOn, Dll4). At the start of any sequence of rules for tip or stalk cell segment movement, the values of these global Booleans dictate whether or not a certain event is permitted. To restrict elongation from occurring during proliferation of stalk cells, and vice versa, there are also local Booleans employed for the individual stalk cells to indicate what event they just performed, and therefore what they can or cannot do in the same and following timesteps.

**Table 4:** Boolean variables determining cell rules.

**Variable**	**Definition**	**Value (Default in Bold)**
ProliferationTipOn	Do tip cells proliferate?	**True **or False

ProliferationStalkOn	Do stalk cells proliferate?	**True **or False

MigrationTipOn	Do tip cells migrate?	**True **or False

ElongationOn	Can cells elongate?	**True **or False

Dll4	Is Dll4 at control levels or is there Dll4 haploinsufficiency?	**0 (control) **or 1 (haploinsufficiency)

**Figure 2:**
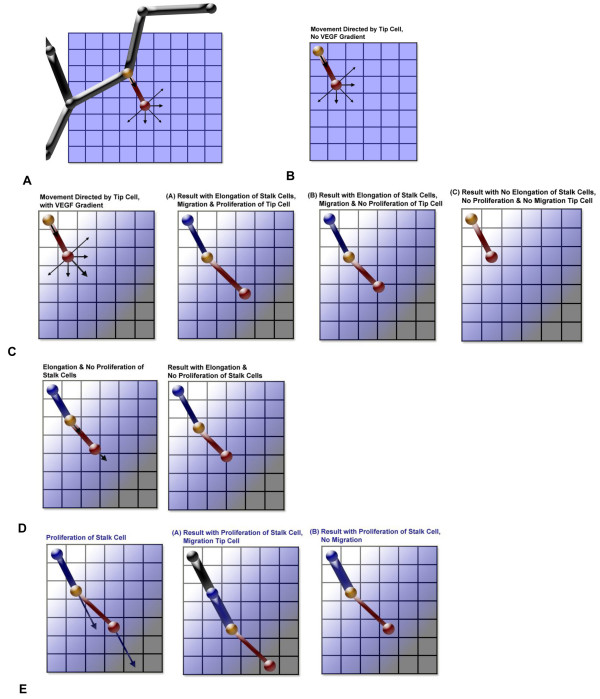
**Illustrations of cell movement represented by rules in the model**. For clarity, movement is shown in two-dimensions. The tip cell is represented by a red node and segment; the node shared between the tip and stalk cells is yellow; and the blue node and segment is the adjacent stalk cell segment. Black segments and nodes represent quiescent vessels. Arrows represent direction of movement for nodes. (A) Schematic of a capillary with an activated tip cell. (B) Movement when there is no growth factor gradient. (C) Movement and the resulting cell segment positions when there is a VEGF gradient, and the effects of allowed stalk cell elongation. (D) Results when there is only elongation of the stalk cell occurring, and no additional migration of the tip cell. (E) Results when there is proliferation of the stalk cells.

Proliferation, elongation and migration of endothelial cells in a growing sprout are represented through the movement of nodes (Figure 2). Throughout the steps in angiogenesis, we focus on three activated nodes representing the tip cell and the adjacent stalk cell segment in every sprout. These nodes are introduced as follows (Figure S1):

(1) Node A, Leading node of the tip cell (red in Figures 2 and S1)

(2) Node B, Shared node of the tip and adjacent stalk cells (yellow in Figures 2 and S1).

This is the back node of the tip cell and leading node of the adjacent stalk cell segment.

(3) Node C, Back node of the adjacent stalk cell segment (blue in Figures 2 and S1)

**Figure 3:**
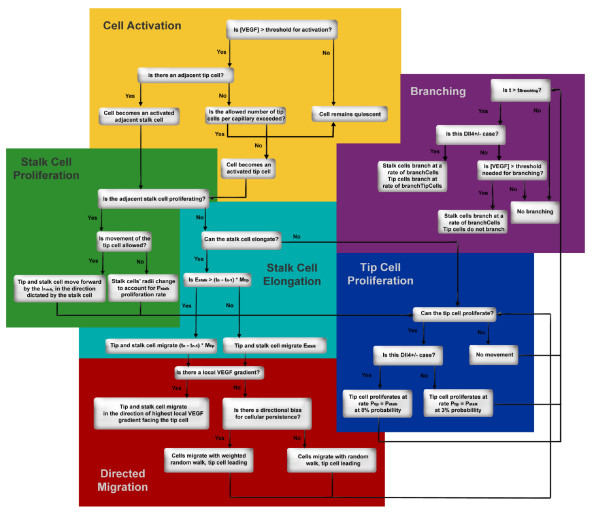
**Flowchart representing the main rules followed by tip and stalk cells throughout a run of the model**. The rules are interconnected, as illustrated by the arrows.

We describe the sequence of events that define the computational processes representing sprout growth, in the context of these nodes. The following paragraphs discuss the processes modeled: cell activation; cell sensing of growth factors; cell migration, proliferation and elongation; cell branching and the process of a sprout joining an adjacent vessel or another sprout. In Appendix 2, we define in more detail events from t_o_, the time at the onset of angiogenesis, to t_n_, a time at any interval following the appearance of a sprout. In Figure 3, we provide a flow chart of the processes to illustrate their connectivity.

### Cell Activation

In experiments of angiogenic sprouting, a single cell is initially shown to branch out in a spindle-shaped fashion from an existing vessel [41-43]. This tapered sprout tip is a highly polarized cell, which expresses genes differently than adjacent stalk cells, including higher levels of VEGFR2 and PDGFβ [1]. The tip cell also proliferates with a much lower probability than stalk cells [1,13] (Table 4).

Cells in an existing blood vessel can be activated by a threshold increase in VEGF protein levels [44]. One cell becomes the tip cell, and a cell adjacent to this tip cell becomes an activated proliferating stalk cell. By secreting matrix degradation proteases like matrix metalloproteinases (MMPs), a tip cell proteolyses its surrounding extracellular matrix and releases matrix-stored growth factors [45,46]. We restrict our initial model to considering the effect of chemical factors on tip and stalk cell response. MMP secretion and matrix degradation are assumed constant. Haptotaxis and the effect of the matrix are represented by adjusting the second term in the cell migration rates, a term that depends on collagen content (Table 3; see Equation S3). The growing sprout, lead by the tip cell, moves along a growth factor concentration gradient, towards the source of higher VEGF. Active stalk cells may change in shape and position, and proliferate, so long as the stalk cell adjacent to the tip cell remains connected to the tip cell throughout.

The computational representation of activation is as follows. An endothelial cell on an existing capillary can be activated in the model when one of its segments is activated. At t_o_, the onset of angiogenesis, there is a search routine over all the cell segments in the model of the existing capillary network. A cell segment is activated when both its nodes sense a level of VEGF above a specified concentration threshold, VEGF_activate (Table S1, see Additional file 1). These nodes are labeled activated nodes. With a certain probability limited by the number of tip cells per capillary, a probability defined by the variables tipNumber and tipNumberFrac (Table S1, see Additional file 1), a sprout may originate from one of these activated nodes. Once a sprout forms from a node, nodes adjacent to it on the existing capillary become inactivated.

### Cell Movement Following Activation

#### Cellular Sensing of Local Growth Factor Gradients

The local maximum VEGF gradient ∇[VEGF]_max _for the leading tip cell node is determined by a local search surrounding the node. For a node at position X(i,j,k), the maximum change in growth factor concentration between its current location and its local environment can be defined mathematically:

(1) ΔCmax⁡⋅η=max⁡i'=i−1 to i+1j'=j−1 to j+1k'=k−1 to k+1⌊ΔCo,i'⋅ηi'+ΔCo,j'⋅ηj'+ΔCo,k'⋅ηk'⌋

**η**_i _is the weighting vector for the +i direction. This weighing vector could be defined as a function of the local matrix, e.g., as a function of collagen fiber orientation. For this model, the matrix is uniform and the weighing vector's magnitude is uniform in all directions and equal to 1. The probability of the cell moving into a potential new position is a function of ΔC, the change in concentration of growth factor between the current position of a cell's leading node (position voxel o), and the highest concentration of growth factor in a nearby voxel (position voxel q, defined by the <i',j',k'> set associated with ΔC_max_), divided by the change in distance between voxel o and voxel q. The larger the positive concentration gradient, the more likely that the cell's node moves towards voxel q. Following this local search routine, the location of the voxel q is where the maximum concentration change per distance was found.

#### Direction of cell velocity (d)

The leading node of a sprout moves in the direction of ΔC_max_·**η**, i.e., **d**(i,j,k) is a function of ∇[VEGF]_max _surrounding the leading node of a tip cell. The local direction for cell movement is recalculated for activated tip cell nodes at each timestep. In the discrete 3D grid used in this version of the model, the directional vector for movement of an active tip cell is defined by changes in the position of its leading node, as follows:

(2) $$\begin{array}{l} d=\frac{{\text{X}}_{\text{o},\text{i}}-{\text{X}}_{\text{new},\text{i}}}{\sqrt{{\left({\text{X}}_{\text{o},\text{i}}-{\text{X}}_{\text{new},\text{i}}\right)}^{2}+{\left({\text{X}}_{\text{o},\text{j}}-{\text{X}}_{\text{new},\text{j}}\right)}^{2}+{\left({\text{X}}_{\text{o},\text{k}}-{\text{X}}_{\text{new},\text{k}}\right)}^{2}}}i\\ +\frac{{\text{X}}_{\text{o},\text{j}}-{\text{X}}_{\text{new},\text{j}}}{\sqrt{{\left({\text{X}}_{\text{o},\text{i}}-{\text{X}}_{\text{new},\text{i}}\right)}^{2}+{\left({\text{X}}_{\text{o},\text{j}}-{\text{X}}_{\text{new},\text{j}}\right)}^{2}+{\left({\text{X}}_{\text{o},\text{k}}-{\text{X}}_{\text{new},\text{k}}\right)}^{2}}}j\\ +\frac{{\text{X}}_{\text{o},\text{k}}-{\text{X}}_{\text{new},\text{k}}}{\sqrt{{\left({\text{X}}_{\text{o},\text{i}}-{\text{X}}_{\text{new},\text{i}}\right)}^{2}+{\left({\text{X}}_{\text{o},\text{j}}-{\text{X}}_{\text{new},\text{j}}\right)}^{2}+{\left({\text{X}}_{\text{o},\text{k}}-{\text{X}}_{\text{new},\text{k}}\right)}^{2}}}k \end{array}$$

Where the designation "new" represents the new X(i,j,k) position determined by the highest local VEGF gradient, and "o" represents the current one. The directional vector is a unit vector, and the local search for maximum concentration gradient is restricted to adjacent voxels. When there is only one direction for the highest VEGF gradient surrounding a voxel, then X_new _corresponds to position voxel q, and **d**(i,j,k) points in the direction of a single ΔC_max_·**η **(Equation 2).

Stochasticity is introduced where the highest gradients are the same in two or more voxels surrounding the sensing cell node, that is, ΔC_max_·**η **and q have more than one voxel associated with them. In this case, **d**(i, j, k) is chosen randomly (with equal probability) between the available locations corresponding to the highest gradient. If all the local concentrations surrounding a cell node are equal in VEGF concentration, the next position is chosen with a bias towards persistence, as described below. If the activated cell is in the position of highest local VEGF concentration, it will continue to move or grow, in a direction chosen from all available locations, also with a persistence bias. The cell searches its local environment and moves based on this weighted set or probabilities, i.e., with a weighted random walk.

#### Persistence

To account for experimentally observed cellular persistence, the probability of moving in specified directions is weighted. Numerous factors could contribute to the underlying biology of observed in vivo persistence, including matrix stiffness, growth factors, filopodia, or directional sensing from a yet uncharacterized source. To analyze the contribution of different factors on biased cell movement, we tested three methods of representing persistence in the model. The first representation of persistence was called intrinsic persistence – a measure of the probability that a cell follows along the same path without deviating direction, independent of growth factors. This was implemented computationally by a rule where a tip cell's leading node remembers its previous location, and a vector is calculated between its previous location and current one. Then the probability of the node moving in the next step along that same vector direction is weighted more heavily; the value of this probability is defined by the variables dirBias and denomBias (Table S1, see Additional file 1). While this is referred to as intrinsic persistence, it is also a means to implicitly represent the process when the leading part of a tip cell alters the local extracellular matrix as it moves (in turn, the extracellular matrix may alter cell integrin binding, signaling and adhesions properties [47,48]) and paves a favorable path for any following cell segments [47,49,50]. A second way of representing persistence was to bias directional movement of cell nodes in favor of a particular location in the entire gridspace. For this representation, movement towards one corner of the grid was weighted more heavily than other directions, and not as a function of VEGF. However, beyond offering a chemotaxic stimulus, growth factors such as VEGF could affect the ability of a cell to follow a given direction [51]. A third representation was weighing directional movement as a function of local [VEGF]. In this implementation, the dirBias variable became a function of [VEGF]. Finally, purely random movement of the tip cell's leading node was compared to the effects of persistence, in the case where local VEGF concentrations surrounding a node are equal.

### Proliferation, Migration and Elongation

The dynamics of proliferation, migration, and elongation in the model represent a novel hypothesis as to how sprout formation is governed by individual cells and cell segment behavior. The result is a push-pull system between tip and stalk cells. As the tip cell migrates out of the existing capillary, it may pull along the stalk cells. This pulling causes the adjacent stalk cell segment to elongate. Diverse stimuli affect elongation of cells during angiogenesis, including growth factors, mechanical stretch and adjacent cells [22,52,53]. We first restrict elongation to result only from a tip cell pulling. Once a tip cell stretches the adjacent stalk cell segment, stalk cell proliferation is stimulated. Stalk cell proliferation in turn pushes the tip cell forward, resulting in tip cell migration. The process then repeats: the tip cell proliferates and migrates towards higher growth factor levels, pulling along the adjacent stalk cell segment, which elongates; then the stalk cells proliferate and push the tip cell forward (for a cartoon representation, see see Additional file 2). Note that the tip cell can also proliferate, with a low probability [13].

In the current model, only the stalk cell segment adjacent to the tip cell elongates, while all the stalk cell segments and the tip cell are able to proliferate. During elongation, the volume of a cell remains constant; the cell radius decreases to compensate for the extended length. The tip cell and stalk cells have a maximum length and volume, which provide limits on velocity and elongation. The following provides the sequential rules that govern the process of angiogenesis after a tip cell appears. The tip cell and the adjacent-to-tip stalk cell segment are the active, moving, growing, and branching segments. The unactivated stalk cell segments in the capillary sprout, all those following the adjacent stalk cell, remain in the position they first establish.

#### Cell proliferation

Proliferation of the tip cell P_tip _is represented in the model by cell volume changes, with time (Table 3). It is defined as the same experimental-based equation for tip cells P_tip _and stalk cells P_stalk _(Equation 10), however stalk cell proliferation occurs deterministically whenever P_stalk _is allowed, while the tip cell proliferation occurs probabilistically as a function of Dll4 (Table 3). Length changes due to proliferation are directly related to volume changes. Following tip cell proliferation, the new radius R_P _and length ℓ of the tip cell are defined by:

(5) R_P _= (cellRadius Fract·P_tip _+ 1)·R

(6) $$\ell =\frac{({\text{P}}_{\text{tip}}+1)\cdot \text{V}}{{\left({\text{R}}_{\text{P}}\right)}^{2}\cdot \pi }$$

where R is the old radius, and V is the old volume of the tip cell segment.

#### Total tip cell movement

The total movement or length displacement of a tip cell, m_total_, at any given time interval t_n-1 _to t_n_, is given by:

(7) $${\text{m}}_{\text{total}}=\{\begin{array}{cc} \min\{({\text{t}}_{\text{n}}-{\text{t}}_{\text{n}-1})\cdot {\text{M}}_{\text{tip}},{\text{E}}_{\text{stalk}}\}+\ell_{{\text{P}}_{\text{tip}}},{\text{where E}}_{\text{stalk}}>0,{\text{P}}_{\text{stalk}}=0 & Case\;1\\ \ell_{{\text{P}}_{\text{stalk}}}+\ell_{{\text{P}}_{\text{tip}}},{\text{where P}}_{\text{stalk}}>0,{\text{E}}_{\text{stalk}}=0 & Case\;2 \end{array}$$

where M_tip _is migration rate of the tip cell. $\ell_{{\text{P}}_{\text{stalk}}}$ and $\ell_{{\text{P}}_{\text{tip}}}$ are length changes of the tip cell due to stalk cell proliferation and tip cell proliferation, respectively. Elongation E_stalk _and proliferation P_stalk _of the stalk cells occur independently and separately, i.e., they do not occur in the same timestep. The specific Cases 1 and 2 refer to when the adjacent stalk cell segment elongates, but the stalk cells do not proliferate (Case 1) and when stalk cells proliferate, but the adjacent stalk cell does not elongate (Case 2). Rules for cell movement in these two cases are described in detail in Appendix 2 (see Additional file 1).

### Branching

Branching occurs at a certain probability after the onset of angiogenesis, and a delay defined by the variable timeBranching. Currently branching occurs with a specified probability for every activated cell during each timestep of the model, if a cell's node senses a specified threshold of local VEGF concentrations (Table S1, see Additional file 1). The minimum branch length is initially set, and the initial angle formed between branching cell segments is also defined, for the current model (Table 2). To visualize the branch (and give a physical dimension to its growth over the 2 hour timestep), we set a minimum branch length that corresponded to at least one grid point away, where growth was allowed in two directions, with a grid size of 1 μm. As an alternative in future models, varying this length, or making the initial branch length dependent on proliferation and migration rates, could be possible. Eventually, the model will include the effect of mechanical forces on cell shape and size; in this case (in combination with any new experimental evidence), we would be able to better justify a range of minimum/initial branching lengths. The branching angle can be randomly selected to be equal, less or greater than this maximum default value, for specific conditions. The presence of Dll4 affects the rate of branching both at the tip and stalk cells; and the effect of Dll4 haploinsufficiency on the branching is represented in the model parameters (Table S1, see Additional file 1). Branching in the model can occur at a stalk or a tip cell node. For a detailed description of how branching is implemented, see Appendix 2 in Additional file 1.

### Joining Another Sprout or Vessel

Anastomoses, or the connection of a growing sprout to another vessel occurs when the leading node of the tip cell touches an adjacent vessel or another tip cell node, randomly. This is a first implementation, and subsequently activation of the adjacent vessel may be a requirement for joining of the tip cell. In this initial model, anastomoses occur infrequently; there is no restriction on sprout movement by surrounding tissue or a bias from interstitial fluid flow.

### Effects of Dll4

The presence of Dll4 has an effect on those rules involving tip cell formation, tip cell proliferation, and branching probability (Table 3). Experimentally, the observed tip cell number in Dll4^+/- ^vasculature is near 1.4 times that of control tip cell number [13]. A two-fold difference was used in the model to allow integer number of tip cells, when observing only a few capillaries. Note also that the number of tip cells formed from an existing capillary versus a new capillary formed by angiogenesis is expected to differ. More tip cell formation and branching is hypothesized in the newly formed vessel.

### Model Parameters

Table 2 lists the parameters in the model, with their relevant references. Table S1 provides initial values of all variables used in the computer code (see Additional file 1). Table S2 provides parameter estimates for cell velocity, as found from in vitro 2D and 3D experiments (see Additional file 1). Velocity values found from Table S1 (see Additional file 1) were used in part to determine migration rules (Table 3, Equation 8; Figure S1, see Additional file 1). Parameters were obtained for endothelial cells where possible. Where a non-endothelial cell type is used, this estimate is stated explicitly. Ranges are given for endothelial cell dimensions in parentheses, and the default values used for this model are presented (Table 2). The value ranges include sizes for mammalian cells, in different tissues. The current model estimates cell and vessel sizes for three-dimensional in vitro conditions, using human umbilical vein endothelial cells. When the model is applied to a specific species and tissue, during certain vascular conditions, the dimensions will be specified for this environment alone.

### Platform

The model was programmed in Java, using Sun's Java3D and MASON (George Mason University, Fairfax, VA, available online: ) libraries. The JAVA IDE used was Borland JBuilder. Output of the Java code (three-dimensional position of the cell segments and capillary structures, and labeling for cell shape and activation status at each timestep) was written into TecPlot (TecPlot, Inc, Bellevue, WA); additional results were written to text files. A description of the computational architecture in provided in Appendix 4 (see Additional file 1). For graphical rendering, the output was read into a user-written POV-Ray program (POV-Ray is available online: ). A POV-Ray movie was then produced from a series of these programs.

### Sensitivity Analysis

Sensitivity analysis was performed for key parameters. Cell displacement rates, [VEGF], migration rates, random weighting, branching probability and timesteps were adjusted over a wide range of values, while other factors were held constant. Sample graphs from the sensitivity analysis are provided in figures, where details of the parameters are relevant to the results and discussion. An exhaustive systematic sensitivity analysis can be performed when the model is restricted to a specific tissue type.

### Timestep

The timestep currently is defined as a constant. As experimental details of events become available, it could be made variable. There is no inherent limit on the number of timesteps.

## Results

The model results show cellular activation, proliferation, and movement during the initial steps in angiogenesis. Four main applications of the model were explored: in silico knockout experiments, characterization of persistence effects on vessel formation, differentiation of tip cell and stalk cell branching, and the effect of Dll4 haploinsufficiency on sprouting.

**Figure 4:**
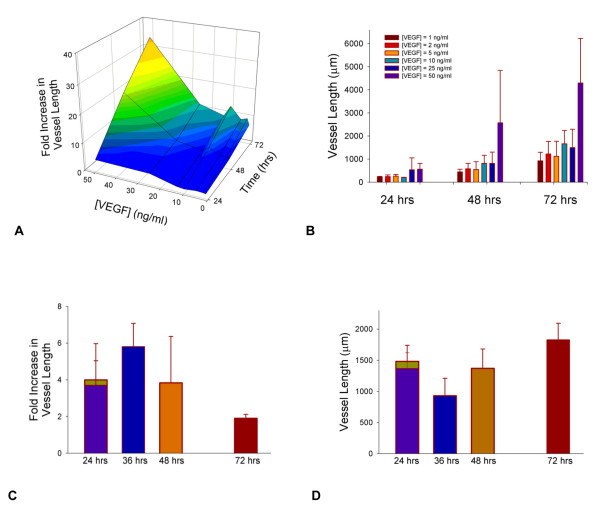
**Relative effect of [VEGF] on total vessel growth over time**. (A) and (B) Effects of [VEGF] alone on total vessel length. Initial number of capillaries was three, and the number of initial sprouts varied from two to six, with branching allowed. Simulation sample size was five values for each concentration at a given time. Growth for this simulation was unrestricted in i- and j-planes, and the dimension of the k-axis was 400 μm. [VEGF] gradients and initial cell activation level ([VEGF] = 0.6 ng/ml) were held constant for all compared [VEGF] concentrations. (C) and (D) Comparison of sprout length changes as a function of VEGF (ng/ml) to experiments using human endothelial cell spheroids on 3D collagen gel. (C) shows fold increase compared to the control in each experiment, while (D) shows absolute changes in vessel length for the same experiments. Values are for growth from a single spheroid. Experiments in references [60-64] were for a mean of 10 spheroids, embedded in a matrix of collagen from rat-tails. Experiments in [60-62,64] used HUVEC alone in the spheroids, while reference [63] used a coculture of HUVEC and human umbilical artery smooth muscle cells. All experiments used 50 ng/ml VEGF_165 _alone as the stimuli, except [61], where 25 ng/ml VEGF_165 _and 25 ng/ml bFGF were added. Experimental data are shown by the purple bar [60], yellow bar [63], blue bar [61], orange bar [64] and red bar [62].

First, to give a basis of how the model's representation of cell activation and chemotaxis correlates with experiments that can serve as validation of the model, cumulative sprout length was compared with and without VEGF at different concentrations. In developing the model, rules for determining the experimental relationship between VEGF concentration and cell migration [54-57] and cell proliferation [44,52,57-59] were estimated from experiments on endothelial cells (Table 3, and Table S2, see Additional file 1). Cell proliferation experiments were 2D in vitro cell culture assays, and cell migration experiments used VEGF as a stimulus for movement of cells in a Boyden chamber assay. In vitro experiments provide an estimate in the computational model for the maximum in vivo changes in response to an activated cell sensing a specified local concentration of VEGF. The effect of VEGF concentration on sprout formation was then predicted by the simulation in three dimensions (Figures 4A and 4B). The model output was qualitatively compared to independent data (data not used in developing the model rules) from experiments on sprout length changes as a function of VEGF in three-dimensional HUVEC spheroid experiments (Figure 4C) [60-64]. Without VEGF or with local VEGF levels less than 0.6 ng/ml, the cells in the model do not become activated, and there are no cumulative sprout length changes.

### Migration, Proliferation and Elongation

**Figure 5:**
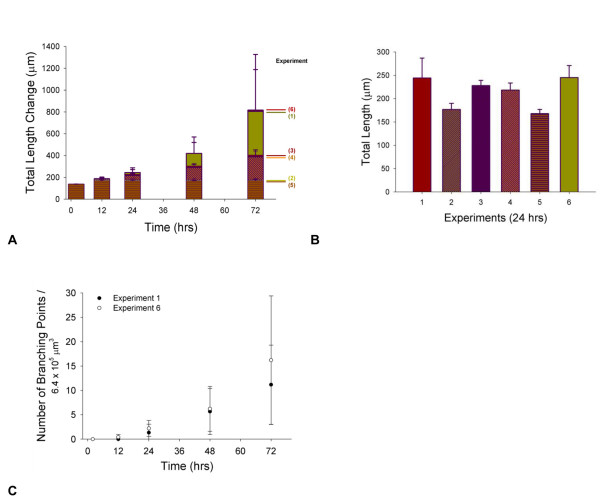
**Results from the in silico experiments**. Total vessel length from 2–72 hrs (A), a snapshot of vessel length 24 hrs after the onset of sprouting angiogenesis (B), and relative branching points over time (C) for the different in silico experimental configurations shown in Table 6. Dll4 = 1 corresponds to wild type, control conditions for this ligand.

Once the premise of cell activation by a threshold VEGF and migration in response to a VEGF gradient was established, varied combinations of the migration and proliferation rules were explored. One highlight of the described modeling approach is that methods were designed with modularity. The effects of allowing or prohibiting processes that would not be feasible to manipulate independently in vivo, can be predicted for specific cell types. Figure 5 shows the effects of altering migration and proliferation differentially in tip and stalk cells, for the Experiments 1–6 detailed in Table 5. There are 32 possible combinations of experiments from manipulating the five Boolean values for initial variables. Boolean values indicate whether or not five processes are allowed (proliferation of the tip cell, proliferation of the stalk cells, migration of the tip cell, elongation of tip and stalk cells, and presence of Dll4 ligand). Initial total capillary length is 127 μm, at 0 hrs, for all model runs. Experiment 5, where no process is permitted, indicates near base line values; in this case, the only growth or cellular addition is the initial formation of tip cells. Experiment 6, on the other hand, stands in for a positive control – all processes are on, and maximum growth and proliferation of both tip and stalk cells are expected. Figure 5C shows how branching is related to elongation and proliferation. Without one or the other, there is no branching (Experiments 2, 3, 4, and 5); significant branching occurs when only tip cell proliferation is turned off (Experiment 1).

**Table 5:** In silico experiments shown in Figure 4.

	**ProliferationTipOn**	**ProliferationStalkOn**	**MigrationTipOn**	**ElongationOn**	**D114**
**Experiment 1**	false	true	true	true	1

**Experiment 2**	true	true	true	false	1

**Experiment 3**	true	false	true	true	1

**Experiment 4**	false	false	true	true	1

**Experiment 5**	false	false	false	false	1

**Experiment 6**	true	true	true	true	1

### Persistence

**Figure 6:**
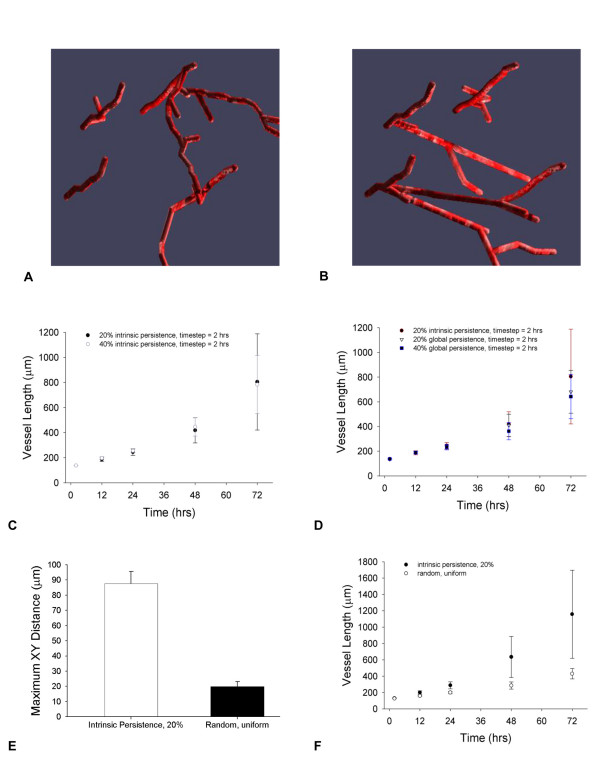
**Persistence comparisons**. (A) through (D) use the default VEGF gradient (Table 3). Visual examples of how directional persistence affects capillary sprout morphology. (A) and (B) show two snapshots of random tip cell movement (A) and 20% intrinsic persistence weighting (B) in tip cell movement, at 48 hours. (C) Total vessel length from 2–72 hrs for the model, comparing 20% to 40% intrinsic persistence. (D) Total vessel length change from 2–72 hrs comparing intrinsic persistence of 20% with global persistence at 20% and 40% directional weighting. For (C) and (D), VEGF concentration is set as a uniform 0.6 ng/ml at each voxel point in the grid, i.e., there is uniform concentration and no gradient. (E) Maximum XY-plane distance reached beyond initial capillary structures for intrinsic persistence weighting of 20% compared to a random weight. (F) Total vessel length from 2–72 hrs for 20% intrinsic persistence weighting vs. random movement.

Representations of persistence were compared graphically (Figure 6). Figure 6 shows the calculations for persistence weighting and the effect on total vessel length over time. When VEGF levels are not uniform, using a search routine for a local gradient (VEGF concentrations directly adjacent to the cell's leading edge) combined with intrinsic cell persistence, produces some random movement of cells (Figures 6A and 6C). When the tip cell's intrinsic persistence is replaced by a biased weight towards a particular grid location, the results are shown (Figure 6B) and compared to length changes in time where there is 20% intrinsic persistence (Figure 6D). With uniform VEGF levels in the grid, entirely random tip cell motion limits the planar-XY growth perpendicular from the initial vessels (Figure 6E), while also limiting the total length of capillary growth (Figure 6F). Without a biased directional preference by cells, the model predicts a tortuous vascular network, regardless of the number or degree of activated cells. Other factors not yet explicitly modeled that could affect persistence include collagen fiber alignment, and the influence of contact guidance [65].

### Branching

**Figure 7:**
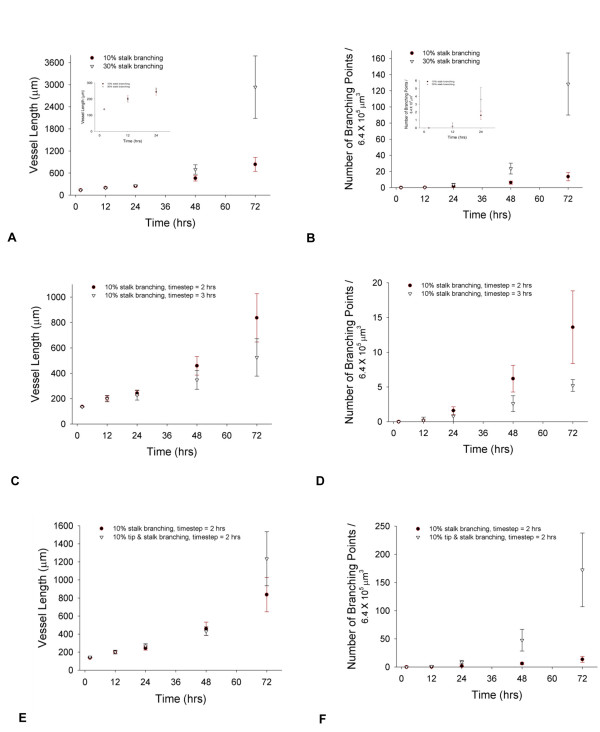
**Analysis of the model's representation of branching for in vitro conditions in three dimensions**. (A) Vessel length over time for stalk cell branching at a probability of 10% and 30%. (B) Corresponding number of branching points for (A). Insets show large scaled values for 2–24 hrs.

The effect of branching on total vessel length is shown in Figure 7. At the onset of angiogenesis, there is little branching; the model predicts it is not until 48 hrs in the default microenvironment, that branching has a significant effect on the total vessel length changes (Figure 7A). Branching predicted by the model correlates to results from available in vitro and in vivo experiments. In HUVECs cultured in 24-well plates with Matrigel (total volume 1.9 ml, 190 mm^2 ^culture area, dimensions found from Corning Cell Culture) at 10,000 cells/well, the number of branch points at 24 hrs ranged from ~5 for cells without growth factor to ~80 for cells cultured with 100 ng/ml soluble Dll4 [66]. Scaling these in vitro findings to the dimensions of 400 μm × 100 μm × 100 μm (dimensions spanned by three initial capillaries and maximum growth), an estimate would be 0–2 branches in 24 hrs, which is what the model predicts at 10% branching for timesteps of 2 or 3 hrs (Figure 7D).

### Dll4

**Figure 8:**
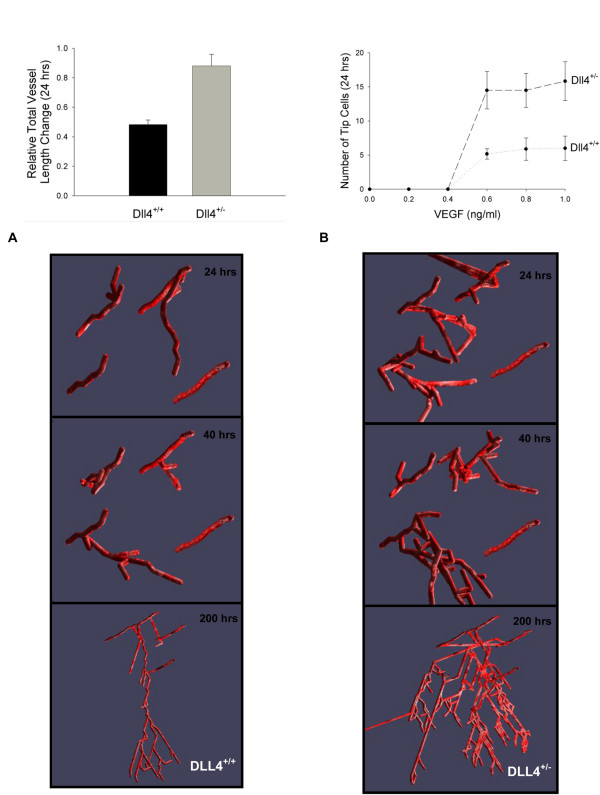
**Effect of haploinsufficiency of Dll4 on blood vessel sprouting compared to control conditions**. (A) Combined effect of [VEGF] and Dll4 haploinsufficiency (Dll4^+/-^) on total vessel growth after 24 hours. (B) Number of sprout tips as a function of [VEGF] and Dll4 haploinsufficiency after 24 hours. For (B), [VEGF] represents both the initial [VEGF], and the [VEGF] used in migration and proliferation rates. Gridspace volume is 1.28 × 10^6 ^μm^3^; initial tips were counted after two hours of stimuli. (C) and (D) Visual snapshots of the model output for control conditions (C) and Dll4^+/- ^(D) after 24, 40 and 200 hrs, with a mean local VEGF concentration above the activation threshold of 0.5 ng/ml. The 24 and 40 hrs runs are in 3D, while the 200 hrs run is shown in 2D.

Figure 8, and Movies 2, 3 and 4 (see Additional files 3, 4 and 5), show the effect of hypothetical haploinsufficiency of Dll4 during sprouting. As Dll4's activity is ingrained in the rules, this is to demonstrate the model's capability to be used as a means to represent phenotypes of in silico knockouts and show the effect of global changes from rules at the single cell and single time-step level. In Figure 8A, the total relative length change in control conditions is compared to conditions where Dll4 is haploinsufficient. The number of sprout tips as a function of VEGF levels in both conditions are compared in Figure 8B.

## Discussion

This model introduces the steps of cellular sprouting during angiogenesis in a manner that is biologically relevant and consistent with experimental observations. Many previous computational models of angiogenesis have been based on equations or rules governing generalized growth factors, without specifying the molecular nature and properties [16,24]. Where VEGF and FGF have been considered specifically, a two-dimensional model provided results with good qualitative agreement with experiments [20]. Models have been deterministic [24] and stochastic, and included differential equations [24], as well as discrete models [21], such as cellular automaton [20]. The agent-based model introduced here offers a three-dimensional, detailed look at the steps in cellular activation, proliferation and movement during angiogenic sprouting. It shows how chemotaxis alone, from a VEGF gradient, affects the velocity and growth of vessel sprouts. The model predicts the degree to which VEGF concentrations beyond an activation threshold influence total vessel length changes (Figure 4), ranks the key individual factors in migration and proliferation by differentiating tip and stalk cell events (Figure 5), explores three potential mechanisms altering cellular persistence (Figure 6), offers one perspective on how branching and vessel length changes could be correlated (Figure 7) and predicts global angiogenic changes to knockout conditions both knockouts at the level of Dll4 receptor binding (Figure 8) and cell-specific processes (Figure 5).

### VEGF Concentrations vs. VEGF Gradients

VEGF is one of the main growth factors involved in angiogenesis. Experimental studies have predicted that the absolute VEGF concentration and the VEGF gradient play separate roles in new blood vessel formation, in a microenvironment-dependent way. The current model represents a situation that might be found for capillary growth in a 3D in vitro setting with human endothelial cells sprouting from an existing vasculature. Perhaps surprisingly, the model predicts little effect of absolute [VEGF] concentration on overall vessel growth in three-dimensions within the range of 1–25 ng/ml (Figures 4A and 4B). At 50 ng/ml VEGF, the average increase in vessel length looks noticeably higher compared to ≤ 25 ng/ml VEGF (Figure 4A, orange and yellow area). However, the degree of variation in vessel length changes is very high at this concentration, and in some instances, vessel length change could be similar to those at lower concentrations (Figure 4B). From visual representations, this phenomenon reflects the possibility of two conditions: one, the initial vessels sprout extensively outward, without physical limitations, and two, the sprouts grow randomly and then back on themselves, limiting total length changes. Thus, from the model, it would be predicted, that where there is no VEGF gradient and cells are activated, total vessel length increases significantly from 24–72 hrs (8× or more), though largely independent of the concentration of VEGF beyond 1 ng/ml; this is in good qualitative agreement with the trend lines for VEGF dependence from experiments using a bovine aortic endothelial cell radial array assay, which showed saturation in vessel length changes with increasing [VEGF] [67]. Furthermore, the model predicts that increasing overall uniform VEGF concentration increases the variability in the length changes. In qualitatively comparing the model predictions to experimental measurements of sprout increase as a function of VEGF and time in HUVEC spheroid models seeded in a collagen gel, two things are of note. One, the variability in results from one experiment to another is high (Figure 4C), while two, the sprout length changes are generally less than that predicted by the model at 48 and 72 hrs (Figure 4D). This latter observation may reflect that the current model has no boundary restrictions. In vivo, the sprouting capillaries would be restricted by tissue and other vasculature, and in vitro, the extracellular matrix could affect growth differently than modeled, as could the limited viability of the cells in culture. In the current simulation, there is no mechanical limitation on their growth and only several initial capillaries present in the model; moreover, cell apoptosis and vessel pruning has yet to be considered. In further renditions of the model, it will be important to test the effect of larger capillary networks; the presence of other cells and tissues; and the effect of apoptosis and vessel pruning.

While independent rules for migration and proliferation are a function of absolute VEGF concentration, the driving force for angiogenesis predicted by the model is VEGF gradient (Figure 4B). The structure of the vasculature in the simulation changes over time, and this is caused by VEGF gradients stimulating a directed growth with the described push-pull phenomenon associated with tip and stalk cells. The VEGF gradient provides a chemical cue that promotes the motion of the tip cell. Increases in VEGF concentration alone do not have this effect, but rather increases speed and random motility of cells, in the model; this has also been supported experimentally [51,68]. Once the leading node of the tip cell moves, the adjacent stalk cell elongates or grows to maintain contact and vessel integrity.

Beyond what has been modeled and discussed so far, there are a number of other factors that influence VEGF gradients and a cell's response to them. Endothelial cells themselves may secrete VEGF as they form a sprout. Outside the primary source of VEGF stimuli, cells such as pericytes, endothelial cell precursors and smooth muscle cells may influence VEGF levels by secretion or physical position. Extracellular matrix heterogeneity and the presence of different VEGF receptor isoforms and heparin binding groups on cells would also alter the response to VEGF gradients.

### Proliferation

We define the probability of proliferation of tip and stalk cells to be different, but the rate of proliferation, once initiated, to be the same. Another way of representing observed differences in cell number between tip and stalk cells, would be to change the rate of proliferation, and keep the probability uniform. The rationale for using the first was that a tip cell has in the past been considered as nonproliferating, leading to a hypothesis that the ability to proliferate increases in different conditions, and that there are subpopulations of proliferating and nonproliferating tip cells.

### Knockout Experiments: Elongation, Proliferation and Migration

In the model, it is predicted that cell elongation has a significant effect on total vessel length (Figure 5B, Experiments 2 and 5, and Table 5). This is because elongation is the stimulus for cell proliferation and migration; without it, the cell may migrate to an extent, but will not proliferate until stimulated. The event knockout experiments of Table 5 and Figure 5 also reflect the extent to which stalk cell proliferation dominates tip cell proliferation. The total vessel changes in Experiments 3 and 4 are nearly identical, where the difference between them is only that Experiment 4 has both tip and stalk cell proliferation restricted whereas Experiment 3 has just stalk cell proliferation restricted. Furthermore, Experiment 1 has a similar pattern of growth to Experiment 6, where again the only difference is the ability of the tip cell to proliferate in Experiment 6.

### Persistence

Without a heavily biased directional preference by cells, the model predicts a very tortuous vascular network (Figure 6). These results were based solely on a search routine for a local gradient (VEGF concentrations directly adjacent to the cell's leading edge) combined with intrinsic cell persistence. This implies that a highly random movement of cells would be present in angiogenesis, even with a local chemotaxic signal, if the extracellular matrix had no effect on cell migration. Alternately, this suggests that the sensing of growth factors like [VEGF] goes beyond local changes, and requires sensing gradients at longer distances than a few microns. If a cell is sensitive to local changes in [VEGF] on the order of microns, the latter hypothesis gives credence to a balance between gradient sensing at a longer distance scale (e.g., by filopodia) and local sensing at the cell surface. It has been shown that retinal endothelial cell filopodia can extend beyond 100 μm, and they are capable of sensing and responding to VEGF via VEGFR2 [1]. Another study indicated endothelial cells can sense directionality in 3D collagen gel for distances of 600–800 μm [62]. These experimental observations support modeling persistence as a function of growth factor concentration as well as a function of the intrinsic probability of following a continuous path in a given direction.

### Delta-like ligand 4 Signaling

Delta-like ligand 4 (Dll4) is a transmembrane ligand for Notch receptors, and it is critical to vascular development. So important is Dll4, that like VEGF, haploinsufficiency of the Dll4 gene is embryonically lethal in many mice strains, as a result of extensive vascular defects [7-9]. Dll4 is primarily expressed in endothelial cells, and correlated to the local concentration of VEGF. The model predicts the effects of a Dll4^+/- ^on the phenotype of new vessel growth (Figure 8). In the Dll4^+/- ^knockout, the vessels show greater total vessel length compared to those in simulated controls (Figure 8; Movies S1 and S2, for control and Dll4^+/-^, respectively). This increase results from a combination of an increase in the total number of sprout tips (Figure 8B), greater tip cell proliferation rates, and greater degree of branching (Movie S3, see Additional file 4) in Dll4^+/- ^vasculature. The absolute magnitude of Dll4 concentration could not be predicted in the current model, based on existing rules (Table 3); these rules can be amended as experimental data becomes available.

It is worthwhile to mention limitations of the current model in resolving existing experimental observations on Dll4 effects during angiogenic sprouting. Studies have indicated an increase in vessel diameter in Notch-inhibited cell cultures [12], while others have shown in Dll4^+/- ^retina microvasculature, vessel size is similar to wild type, and the increase in vessel density is attributed predominantly to increased sprout numbers [69]. The model represents the latter as a first approximation. It may be that both situations occur, depending on the local microenvironment, existing vasculature, and degree of Dll4^+/- ^and Notch expression. Furthermore, here we simplify the system by rules that define the effects of Dll4 haploinsufficiency. In the future, Dll4 expression being induced by VEGF or Dll4 downregulating the expression of VEGFR2 merit consideration [10,70]. Heterogeneity and location of Dll4 and Notch expression among the endothelial cell population may play a significant role in formation of sprouts, and VEGF signal transduction. For example, transiently Notch may be overexpressed by stalk cells, inhibiting the production of new sprouts [71]. Currently, the model limits the number of tip cells present under different conditions (control and Dll4^+/-^), an implicit representation of the signaling that occurs to limit activated cells adjacent to a sprout from also becoming a sprout and disintegrating the integrity of the existing vasculature.

Beyond restrictions in how the model represents Dll4^+/-^, other limitations of the current model warrant discussion. In this model, numerous known biological entities and processes are not considered: hypoxic-induction of HIF1; HIF1-dependent regulation of VEGF; isoforms of HIF and HIF's hydroxylation proteins; VEGF isoforms; a non-uniform extracellular matrix; and matrix-cell interactions, beyond a general representation of how matrix composition contributes to migration (Table 3, Equation 9); VEGF release from the extracellular matrix by endothelial cell-released MMPs; the dynamics of basement membrane deposition around the stalk cell; the effects of parenchymal and stromal cells; and vessel retraction. Experimentally, chemotaxis can generate neovascularization [72], and for the current model, we neglected mechanical factors, including fluid shear stress and explicit changes in matrix stiffness. In future studies, it would be interesting to examine in detail, the contribution of mechanical cues, matrix density and haptotaxis on cell response in angiogenic sprouting, as well as all of the above-mentioned factors that influence angiogenesis.

Other considerations include that capillary lumen and vessel diameter may change in response to angiogenic stimuli, as has been shown in hepatocellular and pancreatic tumors [73]. Assuming that VEGF concentration is constant is an initial simplification; in reality, during angiogenesis, endothelial cells uptake and may produce VEGF.

There also remains a question as to how and when adjacent capillaries connect. In the current model, sprouts or vessels connect when in contact. Further studies should elucidate how the sprout tip of one activates the other, and the degree to which the alignment and anastomosis of the capillaries is driven by collagen fibril alignment [62]. Tip cell filopodia, whose length could be upwards of 100 μm in retinal endothelial cells [1], may sense adjacent vessels and contribute to the formation of capillary anastomoses, as well. In vitro, endothelial cells have been shown to sense collagen gel alignment over 600–800 μm away [62]. Additionally, average capillary length in experiments of angiogenic sprouting is reported with wide variability (on the order of ten microns to many hundreds of microns), and depends highly on the microenvironment [74] and type of endothelial cell.

Another possibility is that Notch-Delta signaling dictates the attachment of sprouting vessels to adjacent vessels. Currently the model includes attachment of one vessel to an existing one, or two sprouts joining, randomly, when they physically touch. It is possible only two activated cells can join. Along with testing out different means of vessel fusion, future model versions would explore the effects of apical-basal cell polarity, the necessary flipping of polarity during sprout formation and alterations in vacuole formation (for a review and schematic, see [71]).

Diverse approaches to computationally representing angiogenesis can yield similar results, particularly in relation to capillary network formation. Examples are an energy-minimizing model [21], a mechanical stimuli-based model, a two-dimensional cellular level model [20] and a generic systems model [75]. The question then arises, how are these models related to one another? The methodology presented here allows different influences on cell growth, proliferation and other cellular processed to be considered explicitly and in accordance with experimental data.

One goal of this cell-based model is to build a general framework that would allow it to be combined with models of intracellular molecular interactions, and membrane receptor-ligand binding and signaling [19,39,76-81]. Molecular models carry the advantage that individual compounds and reactions are often the building blocks for therapeutic development. The cell-level model, in turn, provides other benefits – modeling cell behavior at a level where not all molecular details are known, and when an event (e.g., proliferation, migration as a function of growth factor) or sequence of events governs capillary growth. We further the field of angiogenesis systems modeling by approaching scientific questions from the cellular and tissue level. As the focus of the current study was on the effects of cellular proliferation, migration and elongation on vessel growth – molecular details were not required to answer questions of interest. When tied to the molecular level models, a resulting multiscale simulation would be able to show how molecular interactions influence cellular behavior, which in turn determines tissue phenotypes [82,83].

## Conclusion

In sum, this cell-based in silico model of angiogenesis shows the relationship between growth factor gradients, receptor-ligand presence, cell sprouting, cell migration, cell elongation and cell proliferation in three dimensions. The model shows how representing random movement, persistence by intrinsic means, or persistence by a function of VEGF concentrations alters phenotypic vessel length changes. Furthermore, benefiting from the modularity of the computer methods, we demonstrated the effects of migration separate from proliferation on tip cell and stalk cell movement. Finally, the model represents findings of how Delta ligand changes influence capillary phenotype and presents a three-dimensional framework upon which to test and develop biologically realistic mechanisms underlying blood vessel growth.

## Abbreviations

HIF1: hypoxia-inducible factor 1; PDGF: platelet-derived growth factor; VEGF: vascular endothelial growth factor; MMP: matrix metalloproteinase.

## Authors' contributions

AQ participated in the design of the study and its algorithms, programmed the computer codes, and performed computational experiments; ASP participated in the design and coordination of the study. Both authors read and approved the final manuscript.

## Supplementary Material

Additional file 1 **Appendices 1–4, Supplemental Figures S1-S4 and Tables S1-S2. Appendix 1.** Description of the VEGF gradient; Appendix 2. Description of rules for cell movement; Appendix 3. Methodology for computational analysis; Appendix 4. Computational architecture; Figure S1. Diagram for cell movement; Figure S2. Graphs for parameter calculations for proliferation and migration rates; Figure S3. Computer code hierarchy; Figure S4. Example code for a rule; Table S1. Variables for cell model and their initial values; Table S2. Table of experimental value ranges for cell velocity in 2D and 3D. Click here for file

Additional file 2 **Movie S1.** Cartoon of endothelial cell segment movement. Click here for file

Additional file 3 **Movie S2.** Movie of Dll4^+/+^, control conditions. Click here for file

Additional file 4 **Movie S3.** Movie of Dll4^+/- ^conditions. Click here for file

Additional file 5 **Movie S4.** Movie of Dll4^+/+ ^control conditions in 2D. Click here for file
