# Supplementary material for: Elongation, proliferation & migration differentiate endothelial cell phenotypes and determine capillary sprouting
Source: BMC Syst Biol. 2009 Jan 26;3:13. doi: 10.1186/1752-0509-3-13 (PMC2672076; doi:10.1186/1752-0509-3-13)
Supplement: Additional file 1 — Appendices 1–4, Supplemental Figures S1-S4 and Tables S1-S2. Appendix 1. Description of the VEGF gradient; Appendix 2. Description of rules for cell movement; Appendix 3. Methodology for computational analysis; Appendix 4. Computational architecture; Figure S1. Diagram for cell movement; Figure S2. Graphs for parameter calculations for proliferation and migration rates; Figure S3. Computer code hierarchy; Figure S4. Example code for a rule; Table S1. Variables for cell model and their initial values; Table S2. Table of experimental value ranges for cell velocity in 2D and 3D. [file 1752-0509-3-13-S1.pdf]

Supplemental Material

Appendices for Methods
Appendix 1. VEGF gradient
Appendix 2. Description of rules for cell movement
Appendix 3. Methodology for computational analysis
Appendix 4. Computational architecture

Supplemental Figure Legends S1-S4
Figure S1. Diagram for cell movement
Figure S2. Graphs for parameter calculations for proliferation and migration rates
Figure S3. Computer code hierarchy
Figure S4. Example code for a rule

Table S1. Variables for cell model and their initial values
Table S2. Table of experimental value ranges for cell velocity in 2D and 3D

Supplemental Movie Legends S1-S4
Movie S1. Cartoon of endothelial cell segment movement
Movie S2. Movie of Dll4$^{+/+}$, control conditions
Movie S3. Movie of Dll4$^{+/-}$ conditions
Movie S4. Movie of Dll4$^{+/+}$ control conditions in 2D

Appendix 1. Predefined VEGF Gradient

The entire gridspace is initially populated with a local [VEGF] defined for each voxel location X(i,j,k). This value is uniform except for a restricted volume defined by the variables W1, W2, H1, L1, and L2 (Tables 3 and 4). Within this restricted rectangular volume, the probability density function for [VEGF] in the j-direction is represented by a Gaussian distribution:

$$\phi([\mathrm{VEGF}]_j) = \left\{ \frac{1}{\sqrt{2\pi}\sigma} \exp\left[-\left(\frac{[\mathrm{VEGF}]_j - [\mathrm{VEGF}]_{mean,j}}{\sqrt{2}\sigma} \right)^2 \right] \right. \quad \text{(S1)}$$

where the standard deviation, σ, is a variable defined at each model run. The probability density function is truncated at one standard deviation from the mean in the positive or negative direction. The mean [VEGF] in voxels specified by a given j value (and all i and k values, within the restricted volume) is defined as a function of the j-direction:

$$[\mathrm{VEGF}]_{mean,j} = \left| j \cdot \frac{C1}{g_h} - C2 \right| \quad \text{(S2)}$$

The standard deviation is a function of the mean, $\sigma = C3*[\mathrm{VEGF}]_{mean,j}$ (Table 3). This representation of ∇[VEGF] implicitly accounts for variability in local [VEGF] that may occur due to matrix or interstitial fluid heterogeneity. For subsequent versions of the model, ∇[VEGF] would be defined for a specific tissue and condition of interest.

Appendix 2. Description of Rules for Cell Movement

Events from t_o, the time at the onset of angiogenesis, to t_n, a time at any interval following the appearance of a sprout (Figure S1) At t_o, the onset of angiogenesis, a cell can be activated. In the next timestep t_1, a sprout extrudes from one node (Node B) of this activated cell segment (Figure S1, I). A leading node of the sprout becomes Node A, the leading node of the tip cell. The position of Node A is defined in t_1 as tipMin length away from the existing capillary in the direction of local maximum VEGF gradient.

(1) Case 1, Adjacent stalk cell segment elongates, stalk cells do not proliferate: If a stalk cell does not proliferate during a timestep, but is allowed to elongate, then the tip cell can migrate by a certain amount. The rate of a tip cell's migration rate M_{tip} is defined by experiments as a function of [VEGF], whose value is the parameter VEGF_move (Equation 6; Tables 3 and 4; Figure S2 and Table S1, Supplemental Material). VEGF_move can be specified as a constant or dependent on the local VEGF concentration sensed by a node. For the current model, it is specified as a constant.

$$M_{tip} = T1 \cdot [\text{VEGF (in ng/ml)}] + \text{migNoVEGF} \quad \mu\text{m/hr} \tag{S3}$$

To account for haptotaxis and the effects of extracellular matrix composition, the migration rate is rewritten in the form of Equation (9):

$$M_{tip} = T2 \cdot [\text{VEGF (in ng/ml)}] + T3 \cdot K + \text{migNoVEGFMatrix} \quad \mu\text{m/hr} \tag{S4}$$

where default [VEGF] is the user specified variable VEGF_move (ng/ml), in both Equations (S3) and (S4). Alternately, [VEGF] can be set as the greatest local VEGF concentration the tip cell sees. migNoVEGF and migNoVEGFMatrix are migration rates for endothelial cells (in μm/hr) without the presence of VEGF, and without the presence of VEGF but accounting for matrix presence, respectively.

K is a constant representing the fraction of collagen content in the matrix. For default values (Table 3), Equation 8 is equivalent to Equation 9, where T1 = T2 + T3·K/[VEGF]. At the levels of VEGF explored by the model, the linear form of Equations S3 and S4 approximate observations from in vitro experiments, where VEGF is placed in the cell media (Figure S2B). The form of the equation is consistent with Boyden chamber in vitro migration assays, where VEGF is placed in a top or bottom layer of media and serves as a (stimulant and/or) chemoattractant for the migration of endothelial cells from a cell culture layer plated (with or) above the VEGF source (Figure S2B). The experiments upon which the current default parameters for M_{tip} are estimated, are cited [54, 56, 57]. Upper limits on migration rates were estimated from a series of independent migration experiments in 2D and 3D across different cell types (Table S2). The inclusion of a maximum velocity bounds the maximum effect of [VEGF] on migration at high growth factor concentrations.

Magnitude of migration is determined for a specific timestep, and occurs at 100% if allowed, with a limitation. The total movement of a tip cell due to migration is restricted by the amount of maximum elongation of the adjacent stalk cell segment, E_{stalk}. The elongation constant ε is defined for the tip cell and for an adjacent stalk cell segment in Equation 10, where $\ell_{elongation}$ = length after elongating and ℓ_0 = original length before elongation. The maximum value for ε, ε_{max}, is defined in Table 2. $E_{stalk} = \varepsilon_{max} \cdot \ell_{stalk}$.

$$\varepsilon = \left| \frac{\ell_{elongation} - \ell_0}{\ell_0} \right|$$
$$0 \le \varepsilon \le \varepsilon_{max} \qquad \text{(S5)}$$

When there is elongation and no stalk cell proliferation, the tip cell is pulling along the adjacent stalk cell, in the path it makes. Node B moves in the direction of the previous tip cell segment ***BA***. Node A moves by the amount of M_{tip} or E_{stalk}, whichever is less, in the direction of highest local gradient (Figure S1, III, black dashed arrow).

The rationale for using the previous tip cell segment ***BA*** as a direction for the adjacent stalk cell movement reflects experimental work showing one, endothelial cells possess a degree of persistence, the ability to follow along the same path, and two, routes formed by sprouts are more favorable to the movement of cells than novel paths, due to properties of the matrix and/or a cell's ability to produce its own matrix.

(2) Case 2, Stalk cells proliferate, adjacent stalk cell does not elongate: If during a timestep, the stalk cell segments proliferate P_{stalk}, then the tip cell segment will move in the direction of stalk growth, by the amount of proliferation $\ell_{P_{stalk}}$. The growth rate of activated, proliferating stalk cells as a function of [VEGF] is estimated from experiments (Figure S2A).

$$P_{stalk}(\%\ \text{Cell Proliferation vs. Control}) = P1 \cdot [\text{VEGF (in ng/ml)}] + \text{proNoVEGF} \qquad \text{(S6)}$$

where this rate is a measure of stalk cell volume change, and [VEGF] is defined by VEGF_move (Table 3). Stalk cell volume is determined by adding together the volumes of all stalk cell segments in a given sprout, $V_{sproutStalk}$. When the volume changes, the radii of all stalk cell segments will expand by a specified fraction (cellRadiusFract, Table S1) of this volumetric proliferation rate. New volumes based on the expanded radii are calculated for each stalk cell segment. The combined volume of these segments is subtracted from the total anticipated volume due to growth, $(P_{stalk}+1) \cdot V_{sproutStalk}$. The remaining volume defines the length of the new adjacent stalk cell segment, which has the radius of the previous adjacent stalk cell segment. This new segment grows in the direction of the vector defining the previous ***BA***. This new segment defines the position of new Node B, while the old Node B becomes Node C. Node A, the leading node of the tip cell, moves the length of the adjacent stalk cell segment (as determined by P_{stalk}) in the direction that it is pushed, i.e., towards previous ***BA***.

P_{tip} is the proliferation rate of the tip cell, defined by experiments. Tip cell proliferation can occur at any timestep at a small probability specified by the rules in Table S1 and dependant on presence of

Dll4. If tip cell proliferation does occur, then the tip cell segment's leading node, Node A, moves the amount defined by the other events plus the additional amount of proliferation. It is assumed tip cell proliferation extends the length of the tip cell in the direction of highest local gradient. Proliferation is calculated based on the volume of the tip cell. In the current model for this case, the radius of the tip cell segment may change, with the cellRadiusFract defined in Table S1.

The direction Node A moves due to tip migration or tip proliferation is defined by the local maximum concentration gradient surrounding Node A at t_{n-1}. E_{stalk} is assumed to be in the direction of tip cell migration. The direction Node A moves by stalk proliferation P_{stalk} is defined by the direction of Node B's movement. Node B moves in the direction of the vector defining the line between previous Node A and B (normalized and called ***BA*** here). Node B moves the amount defined by the restriction that the distance between the new Node A and Node B is (previous length of the tip cell segment + $P_{tip,}$ for that timestep). The distance moved by Node B could be solely the amount of stalk cell proliferation; alternately, it could the amount of stalk cell elongation due to tip cell migration. Old Node B becomes Node C. Old Node C becomes a quiescent stalk cell node. This process t_n repeats for t_{n+1} to the end of the simulation.

As stated above and in the text, the leading node of a tip cell determines the direction the cell moves. There are 27 possible locations for a node to move, when unrestricted. To account for the distance differences between adjacent positions and positions at a diagonal, a factor of $(1/2)^{0.5}$ was multiplied by the growth factor concentration values diagonal to the position of interest in the same plane; and a factor of $(1/3)^{0.5}$ was multiplied by the concentration values in the corners of the surrounding 27-unit cube. All other concentrations were multiplied by 1. These values were termed magnitude multipliers. Similarly, the three-dimensional rectangular grid was also accounted for when determinations were made for distance changes due to cell migration, elongation and proliferation in the i, j and k directions.

In all cases, the angle of movement was restricted to avoid nonbiological cell morphology and account for mechanical properties of the cell cytoskeleton limiting angular movements. A leading node

was prohibited from moving into adjacent voxels greater than 90 degrees backwards, where "backwards" was defined by the following node of the segment, a surrogate for the previous location of the leading node.

Calculation of new node position The coordinates of the new position for Node A the leading node of a tip cell are then given by:

$$\begin{aligned} \mathbf{X_q}(i,j,k) = \{ & (\mathbf{X_{o,i}} + \mathbf{d_i} \cdot \mathbf{m_{total}} \cdot \mathbf{magnitudemultiplier}); \\ & (\mathbf{X_{o,j}} + \mathbf{d_j} \cdot \mathbf{m_{total}} \cdot \mathbf{magnitudemultiplier}); \\ & (\mathbf{X_{o,k}} + \mathbf{d_k} \cdot \mathbf{m_{total}} \cdot \mathbf{magnitudemultiplier})\} \end{aligned} \tag{S7}$$

where $\mathbf{d}_i$ represents the change in position from the current node location X_o, to that of the new one $X_{q.}$

Growth of adjacent stalk cell In the second timestep following tip cell activation t_2, what was Node A becomes Node B, the shared node between the adjacent cell front segment and the tip cell segment (Figure S1, II). Node B's position is Node A's previous location plus migration during that timestep in the direction of local highest gradient from old Node A. A new Node A is defined as the leading position of the tip cell segment. This new Node A's position is defined by its distance and direction from Node B. This distance is a length of tipMin plus any distance due to tip cell proliferation, P_{tip}, in the direction of the local gradient facing old Node A. Old Node B from t_1 becomes Node C, the node attached to the original capillary.

Movement and growth of sprout together In subsequent timesteps t_3 to t_n, the activated. Moving nodes are Node A, B and C (Figure S1, III). Those three nodes define the tip cell segment and its adjacent stalk cell segment. The segment between Node A and B is the length of the tip cell. It is defined by the length of the initial tipMin plus any growth allowed by a probability of tip cell proliferation P_{tip}, at every timestep. Minimum length of an initial tip cell is defined by tipMin; maximum length of a tip cell is given by tipMax. If tip cell elongation were allowed in the model, its contribution E_{tip}, would also be added to the length of the tip cell segment. In the described version of the model, the tip cell does not

elongate. At t_n, Node A moves away from its previous position by a distance defined by migration and proliferation of the tip cell segment, m_{total}.

Branching A new tip cell segment can be formed by branching at either a stalk cell node (Node B, Figure S1) or tip cell node (Node C, Figure S1). When this occurs, the process described for t_0 to t_{n+1} repeats with the branch node becoming Node B at t_o. The two differences are that the length of tipMin is replaced by length tipMinBranching and the direction of the branch ***BA*** has to be initially a specified angle (branchAngle) from the sprout trunk. In the current model implementation, there is no branching from Node A.

Appendix 3. Methodology for Computational Analysis

Calculation of Persistence in Cell Displacement For cells moving in vivo, there is a certain probability that once moving, they will continue to follow a straight path in the same direction. This is characterized by persistence P, which is calculated from mean squared displacement $\langle \Delta X^2(t) \rangle$ over time t. The formal definition of the mean square displacement in terms of node coordinates is given by:

$$\left\langle \Delta X^2(t) \right\rangle = \left\langle \left(X_{o,i} - X_{A,i}\right)^2 + \left(X_{o,j} - X_{A,j}\right)^2 + \left(X_{o,k} - X_{A,k}\right)^2 \right\rangle \qquad \text{(S7)}$$

This can be redefined as a function of persistence [51, 96, 101]:

$$\left\langle \Delta X^2(t) \right\rangle = 2 n_d \mu \left\{ t - P(1 - e^{-t/P}) \right\} \qquad \text{(S8)}$$

where n_d is the number of dimensions tracked and μ is the random migration coefficient.

Calculation of Mean Displacement VEGF concentration is present throughout the computational gridspace. As a first step, to generate the dynamics of angiogenic sprouting, we assume uniform [VEGF] in the entire grid, except for a rectangular volume, in which [VEGF] is concentrated in a source at the intersection of the j and k planes (defined mathematically as in Table 3). [VEGF] remains constant in each run of the model, and consumption and release of the growth factor by endothelial cells is neglected.

A cell's velocity **v** can be defined in terms of mean square displacement in one-dimension, where X is the cell's position:

$$\mathbf{v} = \frac{dX}{dt} \qquad \text{(S9)}$$

In three-dimensions, the magnitude of the tip cell velocity v is proportional to mean square displacement, at each timestep:

$$v \propto \frac{\sqrt{\left(X_{o,i} - X_{q,i}\right)^2 + \left(X_{o,j} - X_{q,j}\right)^2 + \left(X_{o,k} - X_{q,k}\right)^2}}{t - t_o} \qquad \text{(S10)}$$

Where $X_{o,n}$ is the initial position of the tip cell's first node, in the n-direction at time t_o, and $X_{q,n}$ is its position at time t, after one timestep. Tip cell velocity can be calculated as a moving average, and summed to give an approximation of cell velocity over time.

Appendix 4. Computational Architecture

The current model consists of ten java programs. Of these, there are several helper programs that are necessary for implementation but are not agent classes: a main controller that runs all the programs and calls methods, a program that defines a gridspace, a program that controls input-output passing, and a program that controls graphics. The other six classes are agents. These are “VolumeEnvironment,” “Cell,” “MammalianCell,” “Endothelial Cell,” “Node” and “Segment.” They can change their environment and respond to local and global rules. They each have a suite of methods. They are organized in the hierarchy shown in Figure S3. Each subprogram may inherit rules. This means the rules governing a particular class extend to the class below it. As an example, an EndothelialCell can access all variables and methods it inherits from MammalianCell, without having this code explicitly programmed in its own class. An example of code implementation for a rule is shown in Figure S4.

Supplemental Figure Legends

Figure S1. Movement of tip cells and adjacent stalk cell segment. Node A (red) s the node representing the leading edge of the tip cell; B (orange and green) is the shared node; and C (blue) is the back node of the activated stalk cell segment. Movement for Nodes A, B and C are defined in the text. tipMin = minimum length of a tip cell. P_{stalk} = proliferation of the stalk cell. E_{stalk} = elongation of the stalk cell. P_{tip} = proliferation of the tip cell. l = length.

Figure S2. Estimation of cell proliferation and migration as a function of VEGF concentration from in vitro experiments. (A) Proliferation function calculation. Percentages represent the amount of increase in cell number compared to control. Open diamonds represent data on HUVEC (data point for 165% cell proliferation, at 13 ng/ml VEGF) and human microvessel endothelial cells (data point for 145% cell proliferation, at 13 ng/ml VEGF) after 72 hrs [57]. Filled diamonds represent data on choroidal endothelial cells after 48 hrs [44]. (B) Migration assays used to estimate the initial model function and constants for endothelial cell migration rate as a function of VEGF. Migration assays use VEGF as stimuli for migration in a Boyden chamber assay. Relative cell migration is defined as the fold change in migrating cell number over time compared to control migration without additional VEGF. Respective references are listed. Lines represent initial estimates for constants in Equation 8; the solid line was used for determining default migration rate parameters (Tables 3 and S1), after also analyzing qualitative results from related experiments using higher concentrations of VEGF. HMVEC: human microvessel endothelial cells; RMVEC: rat microvessel endothelial cells; HUAEC: human aortic endothelial cells.

Figure S3. Computer code hierarchy.

Figure S4. Example code for a rule: Governing Dll4 effects on tip cell proliferation

Figure S1.

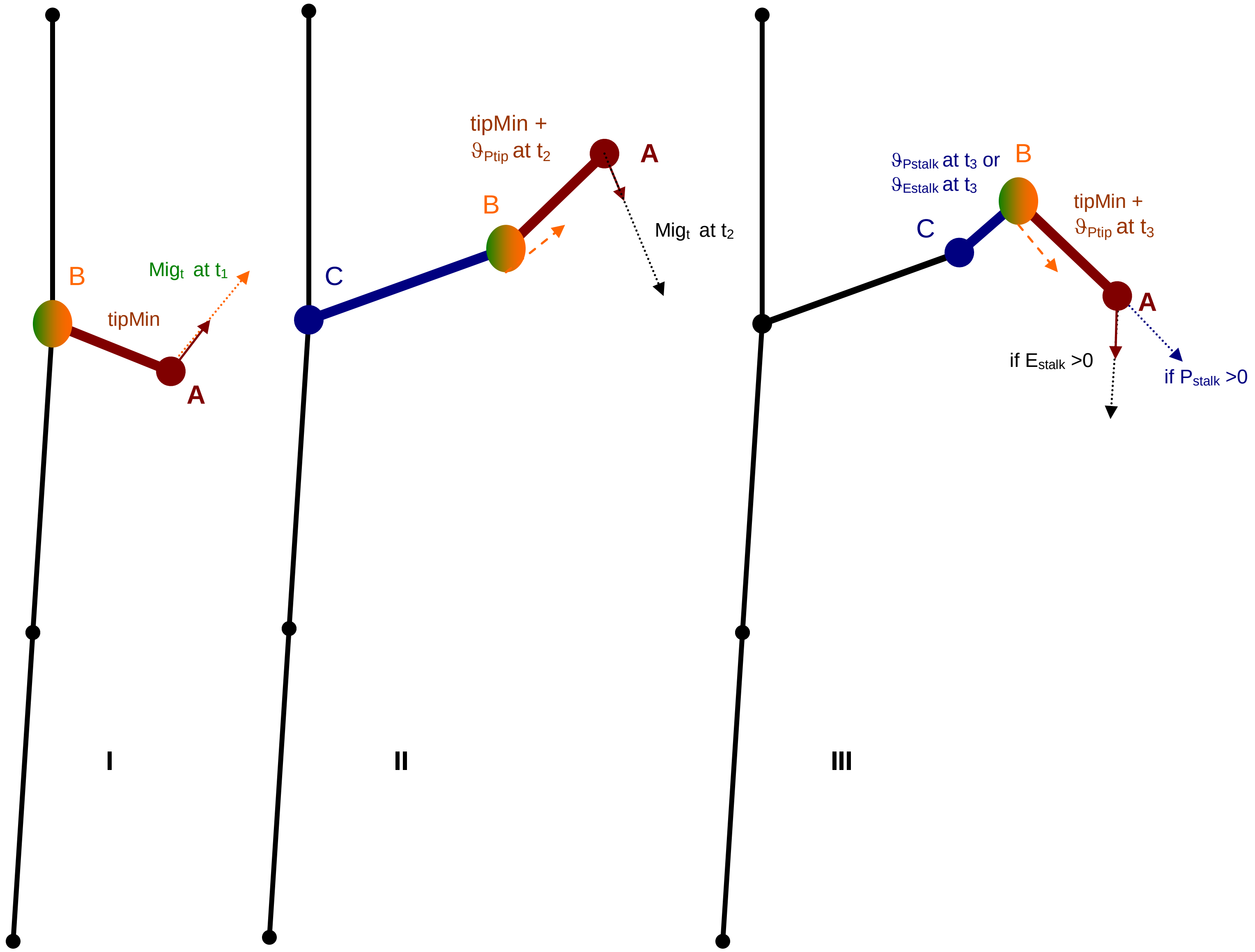
B
Migt at t1
tipMin
A
I
tipMin +
ϑPtip at t2
A
B
Migt at t2
C
II
ϑPstalk at t3 or
ϑEstalk at t3
B
tipMin +
ϑPtip at t3
C
A
if Estalk >0
if Pstalk >0
III

Figure S2.

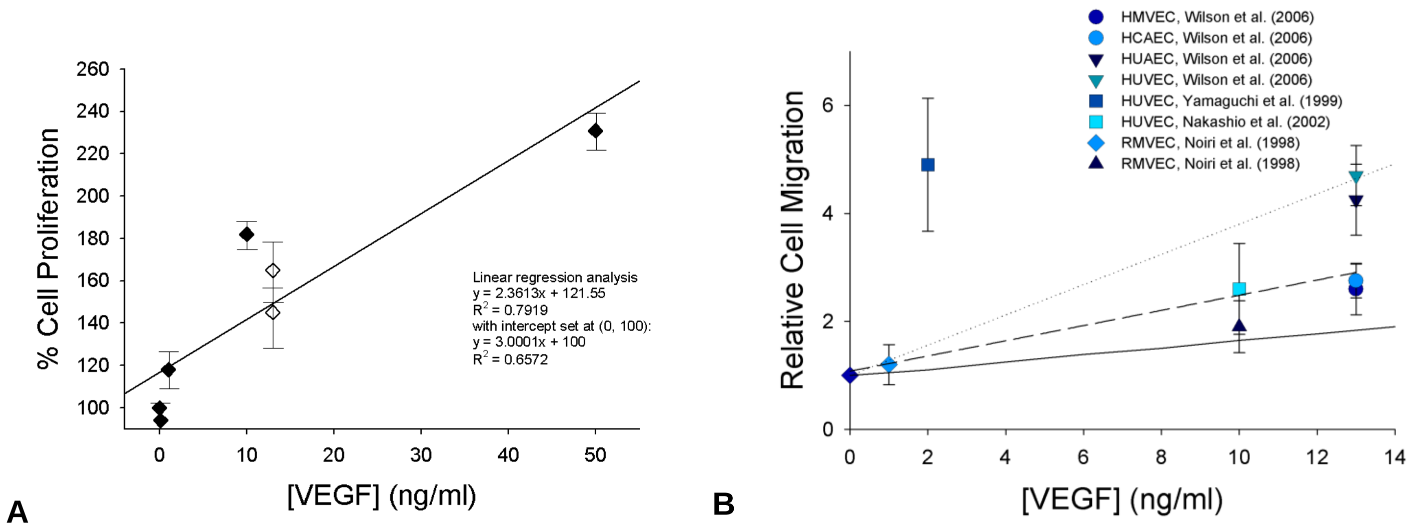
% Cell Proliferation
260
240
220
200
180
160
140
120
100
0
10
20
30
40
50
[VEGF] (ng/ml)
Linear regression analysis
y = 2.3613x + 121.55
R2 = 0.7919
with intercept set at (0, 100):
y = 3.0001x + 100
R2 = 0.6572
A
HMVEC, Wilson et al. (2006)
HCAEC, Wilson et al. (2006)
HUAEC, Wilson et al. (2006)
HUVEC, Wilson et al. (2006)
HUVEC, Yamaguchi et al. (1999)
HUVEC, Nakashio et al. (2002)
RMVEC, Noiri et al. (1998)
RMVEC, Noiri et al. (1998)
Relative Cell Migration
6
4
2
0
0
2
4
6
8
10
12
14
[VEGF] (ng/ml)
B

Figure S3.

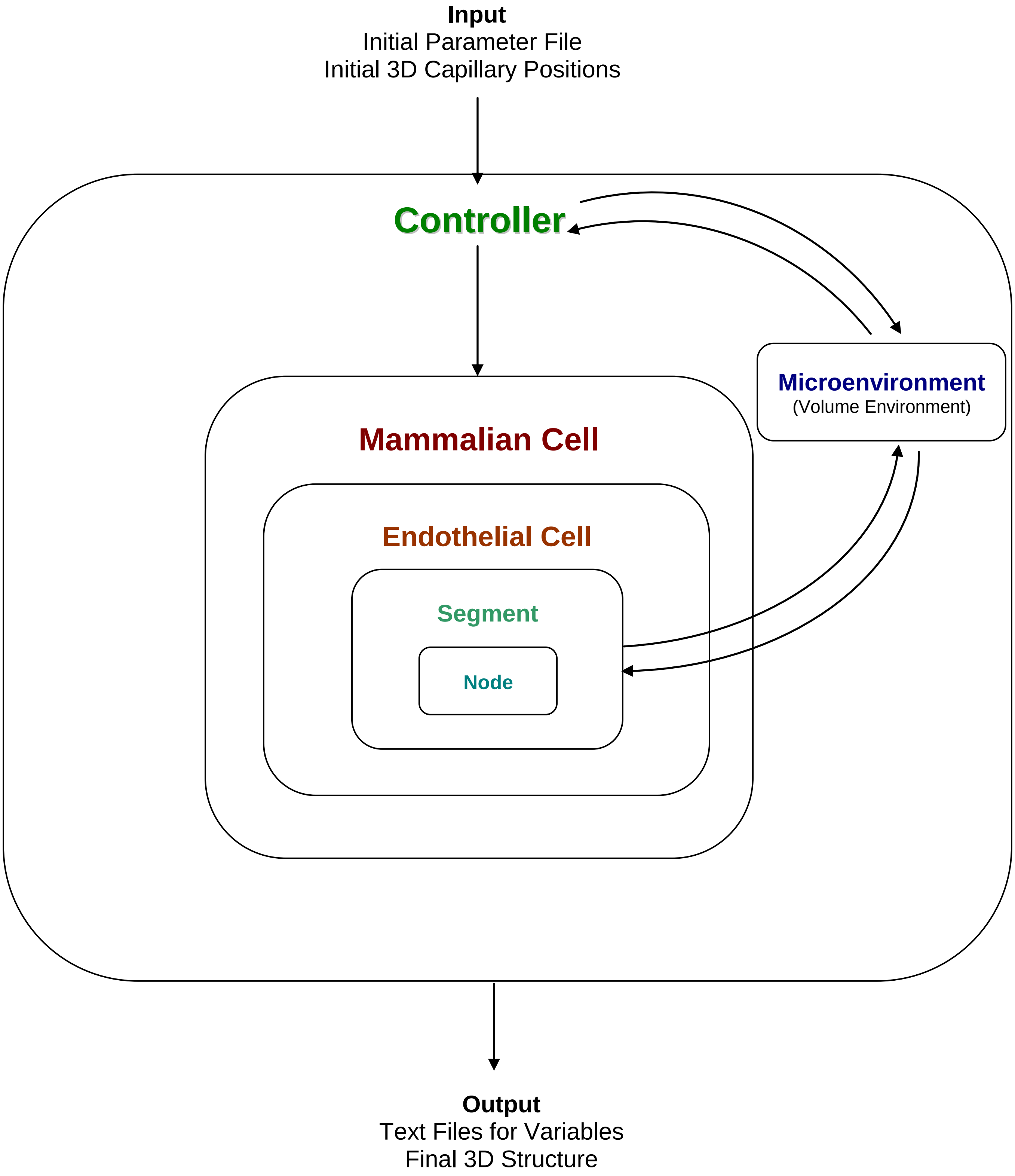
Input
Initial Parameter File
Initial 3D Capillary Positions
Controller
Microenvironment
(Volume Environment)
Mammalian Cell
Endothelial Cell
Segment
Node
Output
Text Files for Variables
Final 3D Structure

Figure S4.

```
if (Dll4 == 1) {
    if (rgen3.nextInt(100) <= P2) {
      proliferationtip = time *
        P1*VEGF + proNoVEGF;
    }
}
if (Dll4 == 0) {
    if (rgen3.nextInt(100) <= P3) {
      proliferationtip = time *
        P1*VEGF  + proNoVEGF;
    }
}
```

If the simulation is of Dll4 wild type
With the probability of 3% (if the next number randomly generated is 0,1,2 of values from 0-99) [13]
then the proliferation of the tip cell is given by experiments for proliferating cells as a function of VEGF concentration.[2]
If the simulation is of Dll4 knock-outs or Dll4 inhibited cells
With the probability of 8% (if the next number randomly generated is between 0-7 of values from 0-99) [13]
then the proliferation of the tip cell is given by experiments for proliferating cells as a function of VEGF concentration.

[2]See Table 4 for references.

Table S1. Variables for the cell model and their initial values.

Variable	Definition	Initial Value	Units
**Local VEGF Levels & VEGF Gradient**			
VEGF_voxel	[VEGF] per voxel	0.6 (default, activated cells)<br>0.1 (inactive cells)	ng/ml
C1	Constant in VEGF gradient	19	ng/ml
C2	Constant in VEGF gradient	4	ng/ml
C3	Constant in VEGF gradient defining standard deviation	0.1	ng/ml
W1	Constant for width division in VEGF gradient	4	dimensionless
W2	Constant for width division in VEGF gradient	8	dimensionless
H1	Constant for height division in VEGF gradient	4	dimensionless
L1	Constant for length division in VEGF gradient	0.25	dimensionless
L2	Constant for length division in VEGF gradient	0.25	dimensionless
VEGF_move	VEGF levels used in calculations for migration and proliferation	0.1 (default, inactive cells)	ng/ml
**Tip Cell Numbers**			
tipNumber	Initial number of tip cells allowed per capillary	1	cells
tipNumberFrac	Fraction difference in number of tip cells per capillary compared to control	2, $Dll4^{+/-}$	dimensionless
**Elongation & Size Changes**			
E_{tip}	Elongation of tip cell	0	μm
E_{stalk}	Elongation of adjacent stalk cell segment	0	μm
M_{tip}	Growth in length for tip cell due to migration	5	μm
m_{total}	Sum of proliferation, migration and elongation for tip cell per timestep	0	μm
tipMin	Minimum length of initial tip cell; tip cell initially grows to this length	5	μm
tipMax	Maximum length of tip cell	60	μm
$\ell_{P_{tip}}$	Change in length due to tip cell proliferation	0	μm
$\ell_{P_{stalk}}$	Change in length due to stalk cell proliferation	0	μm
P_{tip}	Volumetric change in tip cell due to proliferation	0	μm^3
cellRadiusFract	Fraction change in cell radius as a function of cell length change	0.1	dimensionless fraction
**Persistence**			
dirBias	Directional bias; dirBias/denomBias weighs a favored direction out of 8 possible movements when local VEGF levels directly surrounding a cell are equal	> 7 (default), represents 20%, where denomBias = 10, i.e., a random number is generated from [0,10); for the set [8,9] out of 10, move in weighted direction	dimensionless
denomBias	Denominator in directional bias calculation	10 (default)	dimensionless
**Tip Migration**			
T1	Constant in tip migration rule	0.4	μm·ml/(ng·hr)
migNoVEGF	Constant in tip migration rule; cell migration without [VEGF]	6.2	μm/hr
T2	Constant in tip migration rule	0.4	μm·ml/(ng·hr)
T3	Constant in tip migration rule	4	μm/(hr)
T2:T3	Ratio of haptotaxis constant to chemotaxis constant in migration rule	10 (default)<br>0-100 (range)	dimensionless
K	Representation of extracellular matrix; fraction of collagen type I content	1 (represents 10%)<br>0.4 (represents 30%)	dimensionless
migNoVEGFMatrix	Constant in tip migration rule; cell migration without [VEGF] and collagen	4	μm/hr
**Stalk and Tip Proliferation**			
P1	Constant in proliferation rule	2.4	μm^3·ml/ng
proNoVEGF	Constant in proliferation rule; cell proliferation without [VEGF]	121.5	μm^3

	**Dll4 & Tip Proliferation**		
P2	Constant representing degree of tip cell proliferation in Dll4 wildtype	2<br>represents 3%: $[0, 2] \subset 100$	dimensionless
P3	Constant representing degree of tip cell proliferation in $Dll4^{+/-}$ conditions	7<br>represents 8%: $[0, 7] \subset 100$	dimensionless
delta	Delta-like ligand 4 concentration per voxel	0	μmol/ml (ng/ml?)
notch	Notch concentration per voxel	0	μmol/ml (ng/ml?)
	**Boolean Parameters**		
ProliferationTipOn	Boolean defining whether proliferation of the tip cell occurs	1 = true (default)<br>0 = false	dimensionless boolean
ProliferationStalkOn	Boolean defining whether proliferation of the stalk cells occurs	1 = true (default)<br>0 = false	dimensionless boolean
MigrationTipOn	Boolean defining whether migration of the tip cell occurs	1 = true (default)<br>0 = false	dimensionless boolean
ElongationOn	Boolean defining whether elongation of the tip and stalk cells occurs	1 = true (default)<br>0 = false	dimensionless boolean
Dll4	Control or haploinsufficiency for Dll4	1, control<br>0, $Dll4^{+/-}$	dimensionless
	**VEGF Thresholds**		
VEGF_activate	Local [VEGF] threshold required for cell activation	0.5	ng/ml
VEGF_branch	Local [VEGF] threshold required for branching	1 (default, control)<br>0 (for $Dll4^{+/-}$)	ng/ml
	**Branching**		
timeBranching	Time after the presence of a sprouting tip, when branching is allowed	2	hr
branchCells	Probability of a stalk cell branching following 2 hrs of sprout growth.	0.1 (default, control)<br>0.2 (for $Dll4^{+/-}$)	dimensionless fraction
branchTipCells	Probability of a tip cell branching following 2 hrs of sprout growth.	0 (default, control)<br>0.2 (for $Dll4^{+/-}$)	dimensionless fraction
	**Timestep**		
timeStep	Real time equivalent for each computer time step	2	hrs

Table S2. Experimental values of cell velocity in 2D and 3D matrices, with and without growth factors in different cell types.

	Cell Type							
	**MCF-10A human mammary epithelial cells**	**U87-MG human glioblastoma cells**	**Primary human foreskin fibroblasts**	**CCL-209 Bovine pulmonary artery endothelial (BPAE) cells**	**CCL-209 BPAE with 30 ng/ml bFGF**	**Avian primordial endothelial cells**	**Human primary microvessel endothelial cells**	**Human umbilical vessel endothelial cells**
Velocity - 2D	15.3 ± 0.8 μm/hr [102]	31.6 ± 1.0 μm/hr [102]	44.4 ± 3.9 μm/hr [102]	48 ± 1.4 μm/hr [103] (on tissue culture polystyrene)	32 to 46 μm/hr [104]		~25 μm/hr [51] (on tissue culture, calculated by model)	
			34.4 ± 2.4 μm/hr [102]	13 to 34 μm/hr [104] no bFGF			~42 μm/hr [51] (with aFGF, model calculation)	
			41.2 ± 1.4 μm/hr [102]					
Velocity - 3D			25 ± 1.2 μm/hr [102] (on fibronectin in tissue culture dish)					
			34 ± 2.3 μm/hr [102] (on fibronectin in tissue culture dish)	BAEC ~ 4 μm/hr ([62] estimated from experimental calculations, see [45], collagen 1)		5 μm/hr (median), 40 μm/hr (max) [97] (in situ)		~2-5 μm/hr [105] (estimated: Table 1, capillary tube length/24 hr +persistence factor, collagen 1)
